# Modulation of experimental Alzheimer’s disease in rats through donepezil-loaded CSF implant

**DOI:** 10.1038/s41598-025-26181-z

**Published:** 2025-11-25

**Authors:** Lamyaa Osama, Shaimaa ElShebiney, Hanan H. Beherei, Emad M. Elzayat, Mostafa Mabrouk

**Affiliations:** 1 Refractories, Ceramics and Building Materials Dept, National Research Centre (NRC), 33-El Buhouth St, Dokki, P.O. 12622, Giza, Egypt; 2 Narcotics, Ergogenics, and Poisons Department, NRC, 33-El-Buhouth St., Dokki, P.O. 12622, Giza, Egypt; 3 Biotechnology Department, Faculty of Science, Cairo University, P.O. Box 12613, Giza, Egypt

**Keywords:** Alzheimer’s disease, Nanoporous membranes, Drug release kinetics, In-vivo studies, STZ-model, Local drug delivery, Neurochemistry, Alzheimer's disease, Drug delivery, Implants, Nanofabrication and nanopatterning, Alzheimer's disease

## Abstract

Alzheimer’s disease (AD) is a growing challenge worldwide, with current treatments largely symptomatic and limited. The current study aimed to introduce coated nanoporous membranes for localized drug delivery of donepezil, combining both antifouling activity and sustained drug release, unlike traditional Alzheimer’s treatments that rely on systemic administration. Nanoporous membranes were prepared by electrochemical anodization, coated with Polymethyl methacrylate (PMMA) or a PMMA/polyurethane (PU) mixture to mitigate biofouling. The fabricated nanoporous membranes before and after coating were characterized using SEM/EDX, FTIR, BET, and contact angle measurements. In vitro drug release and release kinetics were studied in artificial cerebrospinal fluid (ACSF). Coated and donepezil loaded membranes were implanted on the dura surface in Wistar rats AD model via intracerebral streptozotocin (STZ) injection. The activity was evaluated on behavioral, biochemical and histological levels. PMMA enhanced membrane hydrophobicity (contact angle increased from 62.8° to 79°) and sustained drug release over 7 days, making it the preferred coating. The PMMA membrane demonstrated a reduction in beta-amyloid levels without being loaded with donepezil, while the donepezil-loaded membrane showed cognitive function improvement in Morris water maze and Y-maze. Acetylcholinesterase activity was elevated after STZ-induction of AD and got ameliorated by the membranes implantation. Brain-Derived Neurotrophic Factor (BDNF) was lowered by STZ, while increased in treated animals. Histological examination revealed the neuronal regeneration after donepezil-loaded PMMA coated membrane. These findings suggest that coated nanoporous membranes are promising systems for localized active donepezil drug delivery for AD-like symptoms modulation in STZ-induced AD model, warranting further validation in other AD paradigms.

**Supplementary Information:**

The online version contains supplementary material available at 10.1038/s41598-025-26181-z.

## Introduction

Alzheimer’s disease (AD) is a neurodegenerative disorder marked by the progressive and irreversible decline of cognitive functions, such as memory, language, and learning. Individuals with AD commonly struggle with recognition, communication, speech, movement, and daily activities, resulting in a progressive loss of daily functions. According to the World Health Organization (WHO), dementia affected approximately 57 million people worldwide in 2021. Alzheimer’s disease is estimated to represent 60–70% of these instances. Annually, over 10 million new dementia cases are documented worldwide, a figure anticipated to increase owing to an aging demographic and the persistent absence of viable therapies or prevention strategies^1,2^. This dramatic increase underscores the significant economic and social challenges that AD presents, especially for families in developing and underdeveloped regions.

The blood-brain barrier (BBB) is a selectively permeable structure that controls the transfer of molecules between the circulatory system and cerebral tissue. Although it serves to protect the brain from potentially harmful compounds, it also restricts the delivery of therapeutic drugs^3^. Current oral formulations of donepezil, the primary acetylcholinesterase inhibitor for AD treatment, suffer from significant limitations, including the requirement for high systemic doses to achieve therapeutic brain concentrations, extensive first-pass hepatic metabolism, and dose-limiting peripheral side effects such as nausea, vomiting, and gastrointestinal bleeding that compromise patient compliance and quality of life^4^. Despite advances in nanocarrier-based drug delivery systems, existing approaches still face fundamental challenges including poor control over drug release kinetics, membrane biofouling that reduces long-term efficacy, and lack of sustainable localized delivery directly to brain tissue. One of the strategies employed in this context is nanocarrier-based drug delivery systems which facilitate the transport of drugs across the BBB, owing to enhanced solubility or that they are tailored to release drugs under particular conditions, enabling prolonged drug release, reducing the frequency of doses, and minimizing undesirable side effects. Furthermore, drugs encapsulated in nanocarriers are shielded from enzymatic degradation, which further boosts their effectiveness on the brain^5–7^. A previous study showed that nanoporous membranes with different pore sizes confirmed sustainable drug release and had efficient therapeutic applications^8^.

However, biofouling is considered a major challenge for nanoporous membranes to be applied in brain tissue. The adsorption of proteins and other molecules from the surrounding microenvironment onto the membrane surface, would not only reduce efficiency but also lead to harmful effects such as hemolysis, implant rejection, or infection^9^. Based on current understanding, enhancing the membrane’s surface hydrophilicity and microstructural characteristics appears to be an effective strategy for improving its antifouling properties. Consequently, polymeric coatings have emerged as an effective solution to mitigate fouling effects by serving as antifouling coatings that render the drug carriers more biocompatible and enhance their performance for both diagnostic and therapeutic applications^10^. Polymethyl methacrylate (PMMA) polymer is widely used in multiple pharmaceutical and biomedical applications as for their biocompatibility and minimal tissue inflammatory response, economical cost, feasibility and safety^11^. Another biocompatible polymer is polyurethane (PU), which is essentially used in various industries, including coatings, adhesives, and textiles, in addition to biomedicine^12^. Several reports used functionalized PUs as antifouling agents for drug delivery systems to prevent the adhesion of proteins and bacteria, boosting the performance of these systems^13^. Nishimura et al. patented a medical device containing PU resin elastomer achieving good physical properties and biocompatibility so that it can be used in contact with body fluids or biological tissues^14^. Mixing PMMA with PU offers a unique balance of complementary properties that neither polymer can achieve alone. PMMA contributes rigidity, transparency, and chemical stability, while PU imparts elasticity, toughness, and biocompatibility. Their combination results in a mechanically robust yet flexible composite with improved antifouling performance and tunable porosity. These synergistic characteristics make PMMA/PU blends highly suitable for biomedical applications, particularly in implantable membranes designed for sustained and localized drug delivery^15^.

To address these critical limitations, the present study introduces a novel approach combining anodized aluminum oxide (AAO) nanoporous membranes with dual polymeric surface coatings (PMMA/PU) for direct brain localized delivery of donepezil, thereby circumventing systemic circulation, eliminating peripheral side effects, and achieving sustained therapeutic concentrations at the target site while preventing membrane biofouling through enhanced surface biocompatibility. Accordingly, the present study addressed delivering an acetylcholinesterase inhibitor medicine used in AD, donepezil, locally at microtherapeutic doses by means of a nanodrug delivery system and testing its behaviour in vitro and in vivo.

To achieve this, anodised aluminium oxide (AAO) nanoporous membranes were fabricated and filled with donepezil HCl, then covered with PMMA and PU using a spin coater to minimize membrane blockage and surface contamination, thereby preserving membrane functionality and ensuring consistent drug diffusion in the brain. Developed membranes were characterized before and after drug loading and surface coating using several characterization techniques including SEM/EDX, FTIR, BET surface area and contact angle, then the ability of membranes to release drug before and after surface coating was studied in vitro. Finally, in vivo studies were conducted on Wistar rats with induced AD. The loaded AAO membranes were placed on the dura, and behavioral, biochemical, and histological analyses were conducted at the end of the experiment.

## Materials and methods

### Materials

Aluminium foil (Al, 99.998%, 0.5 mm thick, Alfa Aesar, Ward Hill, MA, USA), acetone (C3H6O, 99.3%, Piochem, Giza, Egypt), sodium hydroxide (NaOH, 98–99%, 39.997 g/mol, El Nasr pharmaceutical chemicals company, Cairo, Egypt), absolute ethanol (CH_3_CH_2_OH, 99.9%, 46.07 g/mol, Merck, Darmstadt, Germany), perchloric acid (HClO_4_, 71–73%, Advent, Mumbai, India), oxalic acid dihydrate (H_2_C_2_O_4_·2H_2_O, 99.82%, 126.07 g/mol, Advent, Mumbai, India), chromium trioxide extra pure (CrO_3_, 99%, 99.99 g/mol, Laboratory Rasayan, Gujarat, India), orthophosphoric acid (H_3_PO_4_, 85%, El Nasr pharmaceutical chemicals company, Cairo, Egypt), sulphuric acid (H_2_SO_4_, 95–97%, Merck, Darmstadt, Germany), copper (II) chloride (CuCl_2_, 99%, 134.45 g/mol, Advent, Mumbai, India), hydrochloric acid (HCl, 37%, El Nasr pharmaceutical chemicals company, Cairo, Egypt), polymethyl methacrylate (PMMA) ( [CH_2_C(CH_3_)(CO_2_CH_3_)]_n_, Mw ~ 15,000, Sigma Aldrich, Darmstadt, Germany), polyurethane (PU) (Sigma Aldrich, St. Louis, MO, USA), tetrahydrofuran (C_4_H_8_O, 99.9%,Cornaredo MI, Italy), donepezil hydrochloride (C_24_H_30_ClNO_3_, ≥ 98% (HPLC), Sigma Aldrich, Lu Wan Qu, Shanghai, China), streptozotocin (C_8_H_15_N_3_O_7_, ≥ 98% (HPLC), 265.22 g/mol, Sigma Aldrich, Saint Louis, MO, USA), citrate buffer (0.1 M, pH 4.5, Biodiagnostic, Egypt), O-acetylcholine iodide (C_7_H_16_INO_2_, 99%, 273.12 g/mol, BDH chemicals, Poole, Dorset, England), 5,5’’-Dithio-bis(2-nitrobenzoic acid) (C_14_H_8_N_2_O_8_S_2_, ≥ 98%, 396.36 g/mol, Corsham, England), ELISA kit for brain derived neurotrophic factor (BDNF) (Elabscience, Wuhan, Hubei, China), Commercial kits from biodiagnostic, Egypt were used for the measurement of MDA, GSH and SOD .

### Fabrication of nanoporous membranes and surface coating

#### Preparation of nanoporous alumina membranes

**Fig. 1:**
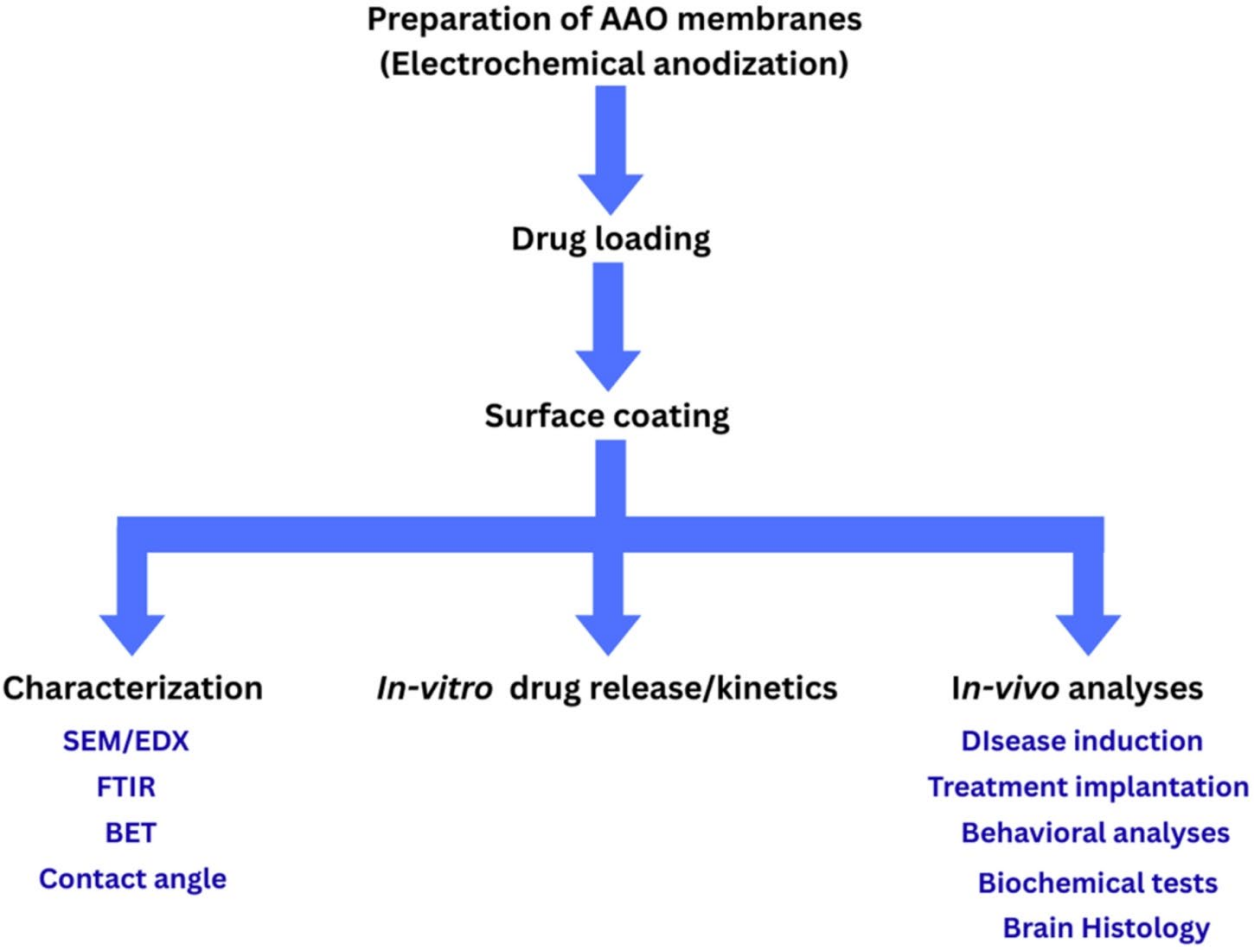
Summary of preformed methodologies in the current research.

High-purity aluminum sheet was subjected to heat treatment in air for 3 h at 500 °C. The surface was subsequently treated with acetone and 1 M NaOH in an ultrasonic water bath for 10 and 3 min, respectively, followed by a thorough rinsing with distilled water. A 1:3 (V/V) mixture of perchloric acid (HClO_4_) and ethanol (C_2_H_5_OH) was employed to electrochemically polish the surface at a voltage of 20 V for 3 min in an ice bath with agitation, as the combination of ethanol and perchloric acid can be potentially explosive, the mixture was prepared and handled with caution in a fume hood. Appropriate personal protective equipment, including a lab coat and acid-resistant gloves, was worn throughout the procedure. The electropolishing step was followed by first anodization in 0.3 M oxalic acid at 40 V for 5 h under cooling conditions with Platinum electrode acting as a cathode and Aluminium sheet as anode. In a 1.8 wt% of H_2_CrO_4_ and 6 wt% H_3_PO_4_ mixture, chemical etching was done at 70 °C for 20 min. Second anodization was preformed exactly as first anodization but for 10 h. At the end of the second anodization, a third anodization was done in 12M H_2_SO_4_ at 40 V for 140 min followed by immersion in etching solution for 1 h at room temperature, in order to separate the AAO membrane from the underlying aluminium. The preformed methodologies in the current research are illustrated in Fig. 1.

#### Preparation of coating materials

Coating of membranes was done using either 5% PMMA only or a mixture of 4% PMMA and 1% PU dissolved in Tetrahydrofuran. Using vacuum spin-coater, 1mL of polymer was added on the top of the membrane, and then it was operated for two runs. The first run lasted 40s at a speed of 500 rpm, while the second run lasted 1 min at 700 rpm. The coating was conducted under ambient conditions. The last step was repeated five times and samples were left to dry overnight. Uncoated membranes and membranes after coating are shown in Fig. S1.

### Characterization of AAO membranes

#### Scanning electron microscopy

The surface morphology and elemental composition of AAO membranes, both before and after drug loading and surface coating, were analyzed using a scanning electron microscope (SEM) combined with energy dispersive X-rays (EDX) (JXA-840 A, Electron Probe Micro-Analyzer, JEOL, Tokyo, Japan) at 15 kV. The membranes were affixed to the stainless-steel sample holders with carbon tape and subsequently subjected to sputter coating with a thin layer of gold. Subsequently, a SEM was employed to analyze the microstructural characteristics.

#### Fourier transform infrared spectroscopy (FTIR)

The functional groups of the nanoporous membranes were examined using attenuated total reflection. Fourier-transform infrared (ATR-FTIR) spectroscopy both before and after drug loading and surface coating both before and after drug loading and surface coating. ATR-FTIR measurements were performed using the FT/IR-4600 type A (Jasco, intensity, Germany) with a scanning range between 400 and 4000 cm^− 1^. The study was conducted at ambient temperature with a resolution of 8 cm^− 1^. Samples were affixed to the specimen container for scanning and subsequently detected.

#### Brunauer–Emmett–Teller (BET) surface area

The Brunauer-Emmett-Teller (BET) method was employed using a Quantachrome Nova 8 Automated Gas Sorption System Version 1.12 to analyze the nitrogen adsorption and desorption isotherms of the membranes prior to and following surface coating, as well as to measure their specific surface areas. The microscopic pore-size distribution at 77 K was determined using indirect molecular adsorption techniques such as N_2_ isotherms and nonlocal density functional theory (NLDFT). Intact membranes pieces weighing 0.2 g were subjected to degassing for 24 h at 150 °C under vacuum conditions. The specific surface areas were computed utilizing the BET method, whereas the total pore volume was ascertained by measuring the gas adsorbed at its highest relative pressure. Values were presented as mean ± SD based on three replicates.

#### Contact angle measurements

The degree of hydrophilicity of the membrane surface specifically indicates the behavior of different foulants upon contact with the membranes. The degree of hydrophilicity of the membrane surface specifically indicates the behavior of different foulants upon contact with the membranes. The contact angle (Θ) is the angle created between the membrane surface and the water droplet interface as the droplet descends vertically onto the membrane surface. The contact angle of both coated and uncoated AAO membranes was assessed utilizing the OCA 15 EC optical contact angle apparatus (Data Physics Instrument, Filderstadt, Germany) in accordance with ASTM-D 7334-08^16^. A droplet volume of 1 µL was used for each measurement. For each group, measurements were performed on three replicate samples, with three measurements taken at different locations on each sample, and values were then averaged.

### In-vitro drug loading and release

500 µg/mL of donepezil HCl were loaded dropwise on 1 cm × 1 cm of uncoated AAO membranes. The drug loading was conducted by adding the whole volume of drug-containing solution to the main sample and leaving it to dry, allowing the membrane to completely adsorb the whole amount. Then membranes were coated with PMMA and PMMA/PU using spin coater (Fig. S2). These membranes were considered to be of an approximate concentration of 5 µg/mm^2^. Drug release efficiency of coated membranes was studied against the uncoated ones. Samples were soaked in 50 mL of ACSF for 14 days and agitated in a shaking incubator at 36 °C with a speed of 50 rpm. During the two weeks of the experiment, 3 mL of the solution were being withdrawn and substituted with another 3 mL of fresh ACSF to maintain sink conditions at designated time intervals of 1, 3, 5, and 24 h, as well as 3, 5, 7, and 14 days. The amount of donepezil HCl released was quantified using UV spectrophotometer at a wavelength of 230 nm^17,18^. The calibration curve used is shown in Fig. S3, with a linearity range from 2 µg/mL to 10 µg/mL, while the possible interference between the coating material and the drug and its effect on the drug release profile are investigated in the following sections.

### Drug release kinetics

The release data was analyzed using zero-order, Higuchi diffusion, and Korsmeyer–Peppas mathematical models to ascertain the mechanism of donepezil release in vitro^19–21^. Using Eqs. (1), (2) and (3), as follows:

1 $${Q_t}=~{Q_0}+{K_0}t~~$$

2 $$Q={K_H}{t^{\frac{1}{2}}}~$$

3 $$\frac{{{Q_t}}}{{{Q_\infty }}}={K_k}{t^n}~$$

Where Q_t_ is the cumulative drug released in time t; t is the leaching time in hours; Q_0_ is the initial drug concentration in the sample; K_0_, K_H_, and K_K_ are the zero-order, Higuchi and Korsmeyer–Peppas dissolution rate constants, and n is the kinetic exponent. A value of *n* < 0.5 indicates quasi-Fickian diffusion; Fickian diffusion = 0.5; *n* = 0.5–1.0 indicates non-Fickian or anomalous transport; *n* > 1.0 indicates Case II transport.

For 50% release (t_50_) and 90% release (t_90_), the following equations are used for zero-order and Higuchi model, respectively:

4 $${t_{t50}}=\frac{{50 - intercept}}{{{K_0}}}$$

5 $${t_{90}}=\frac{{90 - intercept}}{{{K_0}}}$$

6 $${t_{50}}={\left( {\frac{{50 - intercept}}{{{K_H}}}} \right)^2}~$$

7 $${t_{90}}={\left( {\frac{{90 - intercept}}{{{K_H}}}} \right)^2}~$$

### In-vivo study design

#### Animals and experimental design

Male Wistar 10 weeks old rats, weighing 250–300 g, were housed under conventional laboratory conditions (12 h light–dark cycle, 55 ± 2% humidity, and 22 ± 2 °C ambient temperature). The animals had unrestricted access to food and tap water. A total of 48 rats were randomly allocated into eight groups (*n* = 6 per group). For all surgical groups involving membrane implantation, a 20 mm² membrane was placed on the surface of the dura. The groups were defined as follows:

1. **Control group** – Normal rats with no surgical intervention.
2. **Sham group** – Rats underwent the surgical procedure without injection or membrane implantation.
3. **PMMA group** – Surgical procedure with implantation of a PMMA-coated membrane.
4. **AAO group** – Surgical procedure with implantation of an uncoated AAO membrane.
5. **STZ group** – Alzheimer’s disease (AD) model induced via streptozotocin (STZ) injection; no membrane implanted.
6. **STZ + PMMA group** – AD model with implantation of a PMMA-coated membrane.
7. **STZ + PMMA-DPZL group** – AD model with implantation of a PMMA-coated membrane loaded with donepezil.
8. **STZ + DPZL group** – AD model with implantation of an AAO membrane loaded with donepezil.

All experimental techniques or methods involving animals were conducted in accordance with ARRIVE guidelines for animal use and care and received approval from the National Research Centre Medical Research Ethics Committee *(Ethical approval no. 113042023).* All experiments were performed in accordance with the relevant guidelines.

#### Surgical procedure

To prepare for the surgical procedure, rats were fasted 10 h at least before the surgery day but allowed free access to water. For induction of AD in rats, STZ was prepared in citrate buffer (0.05 M) and 2µl was introduced intracerebroventricularly (icv). Under sterile conditions, rats were deeply anesthetized by intraperitoneal injection of ketamine (90 mg/kg) and xylazine (80 mg/kg) mixture, and were placed in a stereotaxic apparatus, a heating lamp was on during the surgery to keep the animal warm, and ophthalmic ointment was applied to prevent corneal drying. The area designated for the procedure was shaved and cleaned with povidone-iodine alcoholic solution and a midline incision of the scalp was done, then the skull was exposed by careful retraction of skin and muscles. Two tiny holes were drilled using electric burr. The injection needle was placed and directed at the lateral ventricles coordinates: -0.8 mm in the anteroposterior axis, ± 1.4 mm in the mediolateral axis and − 3.6 mm in the dorsoventral axis connected to an infusion pump to inject at a rate of 1 µl/min^22^. After injection, the syringe was held still for one more minute, and then same procedure was repeated to the other lateral ventricle of the brain. A 20 mm^2^ piece of each uncoated or coated AAO membrane was then placed just on the surface of the dura for the relevant group, the hole was left without cement, saline-washed then incision was sutured. Sham rats were i.c.v. injected with citrate buffer (2 µl) instead of STZ. Rats were treated after the surgery with antibiotic and analgesic for post-operative care under supervision of the animal house veterinary specialist and allowed to recover for 3 days^23^.

**Fig. 2:**
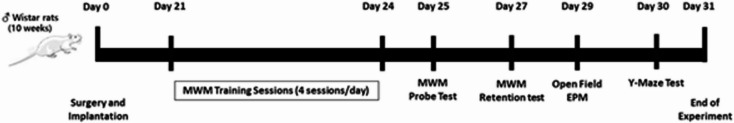
Timeline of the in vivo experimental design.

The study timeline and design is presented in Fig. 2.

#### Behavioral procedures

Two different experimenters than the ones who performed the surgery participated in all behavioral assessments and the groups were coded anonymously for the experimenters.

##### Y- maze test

Y-maze spontaneous alternation is used to evaluate the spatial cognitive power represented in the short term working memory of the animal. The maze was constructed from white laminated wood. Each arm measured 40 cm in length, 15 cm in width, with walls 30 cm in height, and included a triangular middle section ^24^. The three arms are identified as the start arm, the other arm, and the novel arm. On the 30th day post-implantation, spatial recognition memory was evaluated by observing spontaneous alternation behavior in a single-session Y-maze. The session comprised a training trial and a test trial (Fig. S4a). During the training trial, the novel arm was obstructed, and the rats were positioned in the start arm, let to explore the maze for 10 min. Following a one-hour break, the novel arm was opened for a test trial, and the rats were returned to the start arm to freely explore all three arms for ten minutes. The proportion of the new arm to all arms for time spent (time index) and the number of entries (entries index) was documented. Spatial working memory was evaluated by permitting rats to freely explore all three open arms for 8 min the next day. The alternation index has been calculated. The device was sanitized with 70% ethanol between rat experiments.

##### Morris water maze test

Morris Water Maze is a valuable tool to assess cognitive learning and memory implicated by the hippocampal circuitry, where probe test assesses the learning outcome while the retention test evaluates the long-term memory. A circular tank, measuring 130 cm in diameter and 50 cm in height, with a smooth inner surface, was utilized. The pool contained opaque water at a depth of 30 cm, with a temperature of 23 ± 2 °C. A white platform, measuring 10 cm in diameter, was submerged 1.5 cm beneath the water surface and positioned at the midway of a stationary quadrant. In this experiment, each rat underwent training four times daily for four successive days, followed by the test trial on the fifth day. During the training, the animals were randomly assigned to one of the four quadrants of the pool (northeast, northwest, southeast, and southwest) and tasked with locating a platform submerged 1 cm below the water surface in the northeast quadrant within 60 s. Upon reaching the platform, they were permitted a 15-second respite (Fig. S4b). If they were unable to locate the platform, they received guidance. Subsequent to each session, rats were towel-dried and positioned in a lamp-heated enclosure for a minimum of 15 min. During the test session, the swimming patterns (path length) to the target area, as well as the time taken to reach the platform, were assessed and documented. A memory recall test was conducted on the seventh day by eliminating the platform, permitting each rat to swim freely for 60 s, and documenting the duration spent in the platform quadrant^25^.

##### Open field test

Rat locomotor activity was recorded in a five minutes session in a 100 × 100 cm white-walled box. The number of crossings (the number of squares transverse, horizontal activity) and number of crossings to central zone were subsequently recorded after each rat was placed in the center of the field (Fig. S4c). The field was cleaned with 70% ethanol in-between rats^26^.

##### Elevated plus maze test

Rats were tested in a two-arm plus-maze for five minutes. In the center zone, each rat was released precisely in front of closed arms (Fig. S4d). Both the number of entries and the total duration of time spent in closed arms were recorded^27^. The test is an indicator of anxiety-like behavior which is a common symptom of Alzheimer’s disease particularly in the early stages^28^.

#### Brain isolation and biochemical measurements

After the behavioral analyses, blood was collected, the brains of rats were removed, and hippocampi were isolated, blotted dry, weighed, and subsequently prepared as a 10% homogenate in ice-cold phosphate-buffered saline solution (pH 7.4), centrifuged (1000xg, 4 °C, 15 min), and stored at − 80 °C for further experiments. Other brain tissues were fixed in 10% formol saline for histological studies.

##### Measurement of acetylcholinesterase (AChE)

Serum AChE activity was assessed using the colorimetric technique established by Ellman at 412 nm. The assay solution comprised 0.1 M K/Na phosphate buffer at pH 7.5, 25 °C, 0.33 mM DTNB, 0.02 unit/mL AChE, and 1 mM substrate ACh. Reagent blanks comprised reaction mixtures without any of substrates.

##### Estimation of brain-derived neurotrophic factor (BDNF)

ELISA kit for BDNF and was used for hippocampal measurement following manufacturer’s instructions (Elabscience, China).

##### Assessment of oxidative stress markers

Oxidative stress markers were measured in hippocampal homogenate using commercial local kits (Biodiagnostic, Egypt), concentration of malondialdehyde (MDA) was measured at 532 nm according to Uchiyama and Mihara 1978, reduced glutathione (GSH) was determined spectrophotometrically according to Beutler et al. (1963) method using Ellman´s reagent at 412 nm and finally, the activity of superoxide dismutase (SOD) was quantified as previously documented by Bagheri et al. at a wavelength of 550 nm^29–32^.

#### Histopathological examinations

After fixation for 48 h at least, tissue were alcohol-dehydrated, and embedded in paraffin blocks. Hematoxylin and Eosin (H&E) and alkaline Congo red to visualize β-amyloid fibrils were used to stain tissue sections for histological evaluation under Leica optical microscope and image analysis system. Evaluation of Congo red stain intensity was quantitatively assessed by a histomorphometric analysis using Image Analysis System at the Pathology Laboratory, at the Medical Research Centre of Excellence (MRCE) unit, National Research Centre using the image analysis system Leica Qwin DW3000 (LEICA Imaging Systems Ltd, Cambridge, England), which consists of Leica DM-LB microscope with JVC color video camera attached to a computer system. The degree of reaction was chosen by the color-detect menu to show the degree of intensity. A total of five field measurements were taken per slide in brain tissue stained with congo red stain.

#### Statistical analysis

Data were analyzed for differences with one-way ANOVA. The data underwent a Kolmogorov-Smirnov analysis to assess normality of distribution. ANOVA was followed by a post-hoc evaluation employing the Tukey test. Non-parametric data were evaluated using one-way ANOVA Kruskal-Wallis, followed by the post hoc Dunn test. All data were presented as means ± standard deviation (SD). Differences were deemed significant at a 95% confidence level when *p* < 0.05.

## Results and discussion

### Characterization of AAO membranes

#### Scanning electron microscopy (SEM)

**Fig. 3:**
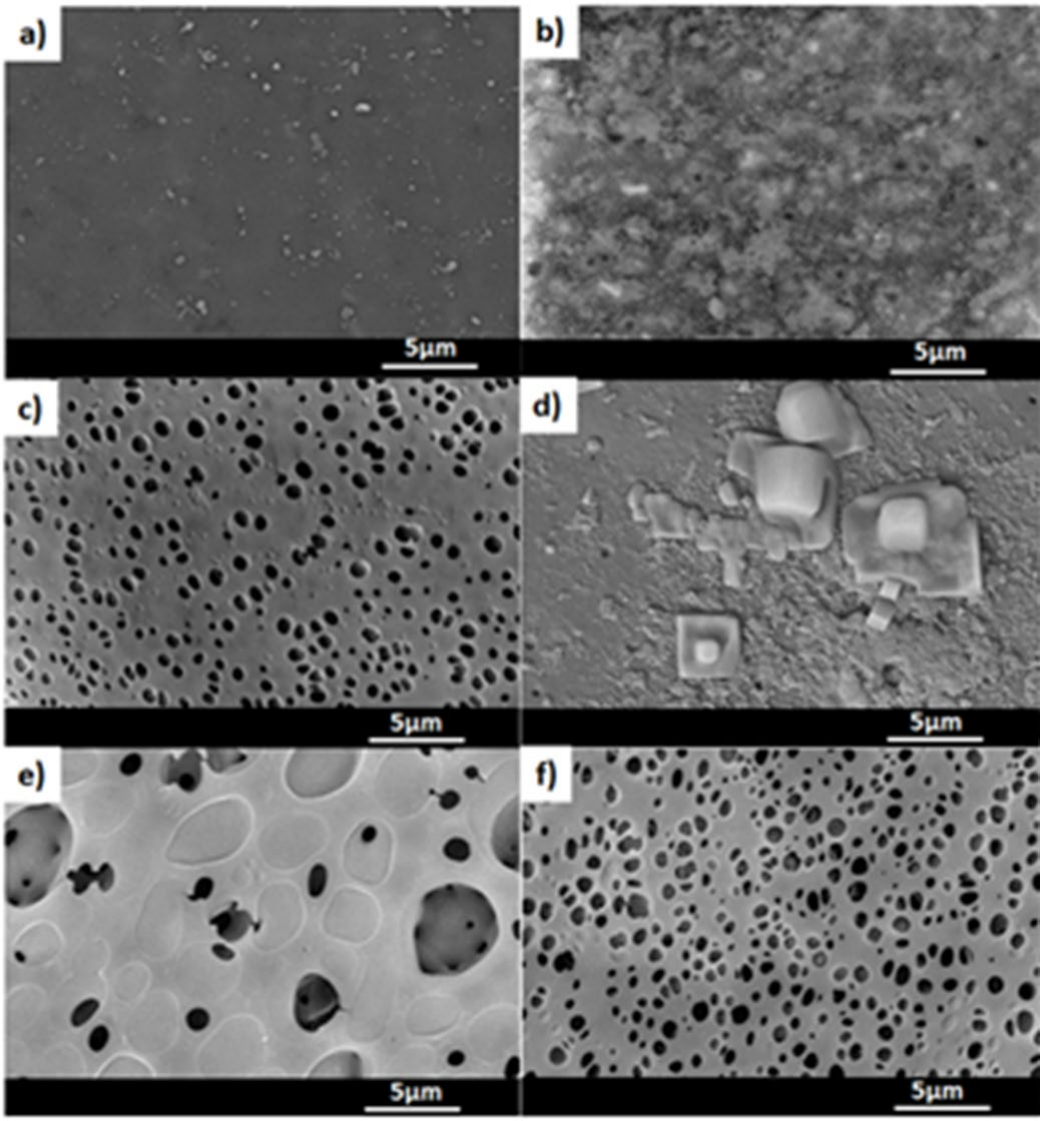
SEM images of (a) AAO membrane, (b) AAO membrane loaded with Donepezil, (c) PMMA coated membrane, (d) PMMA coated and Donepezil loaded membrane, (e) PMMA + PU coated membrane and (f) PMMA + PU coated and Donepezil loaded membrane.

**Fig. 4:**
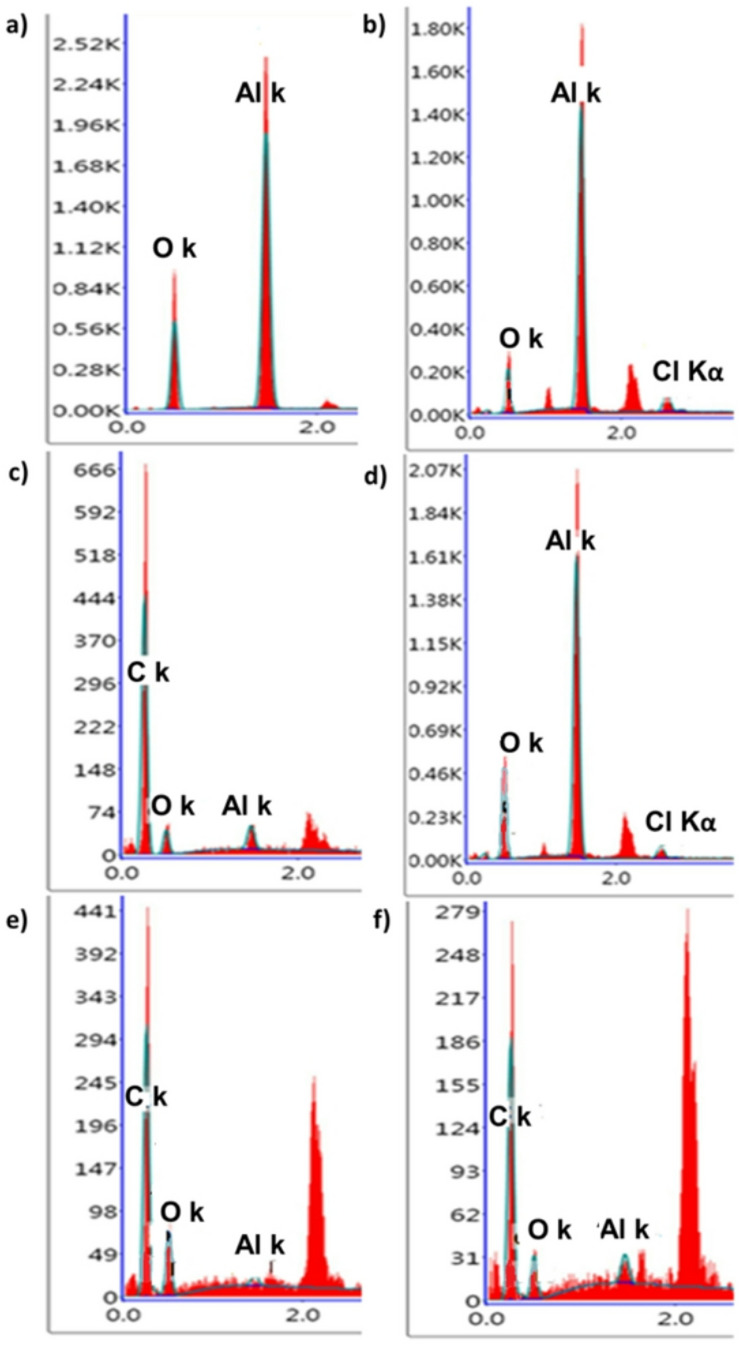
EDX charts of (a) AAO membrane, (b) AAO membrane loaded with Donepezil, (c) PMMA coated membrane, (d) PMMA coated and Donepezil loaded membrane, (e) PMMA + PU coated membrane and (f) PMMA + PU coated and Donepezil loaded membrane.

Figure 3 shows the obtained AAO membranes before and after drug loading and surface coating. The obtained AAO membranes have shown a homogenous porous surface (Fig. 3a) and the purity of the membranes was confirmed by the presence of Al and O shown in the corresponding EDX (Fig. 4a). Donepezil-loaded membrane showed no agglomeration of drug particulates on its surface that suggests the presence of drug inside its channels or attached to the surface (Fig. 3b). In addition, EDX confirmed the presence of drug residues on the surface by the existence of Cl in the chart (Fig. 4b). After coating with PMMA, a thin layer of polymer appeared to have pores and the presence of C in EDX confirmed the successful coating with PMMA (Figs. 3c and 4c). Loading the membranes with Donepezil and then coating with PMMA is shown in Fig. 3d that revealed the presence of Donepezil particles embedded within the polymeric layer as Cl appeared in the corresponding EDX chart (Fig. 4d). Coating with PMMA and PU offered a denser surface coating morphology (Fig. 3e) with an increased C content due to presence of both polymers in EDX (Fig. 4e). The presence of drug beneath the polymers caused an increase in the porous morphology of the polymeric layer (Fig. 3f). This was confirmed by EDX analysis (Fig. 4f), as the absence of Cl indicated that the polymer coating completely covered the drug.

#### FTIR of membranes

**Fig. 5:**
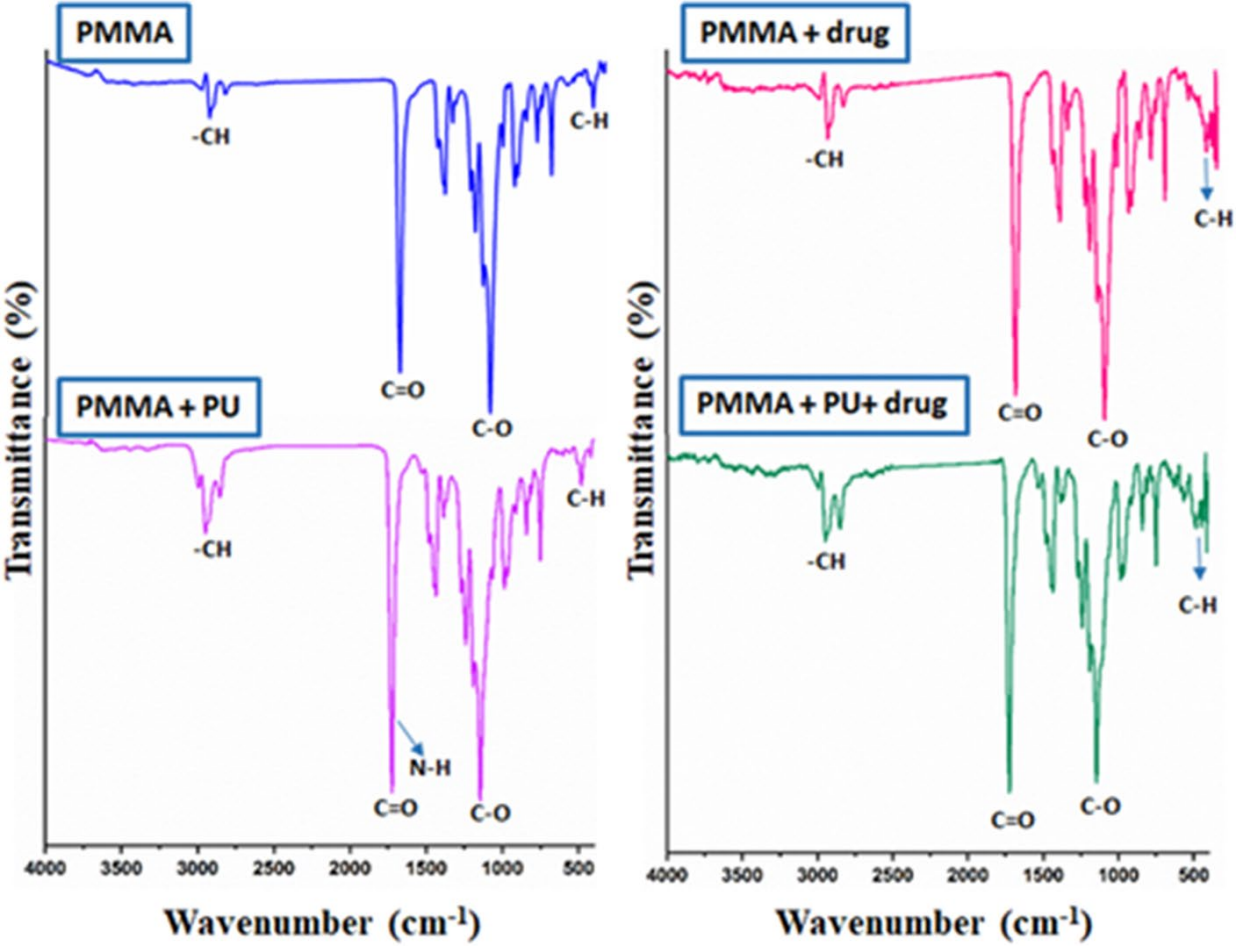
FTIR results of coated membranes before and after drug loading.

FTIR analyses of coated AAO membranes before and after Donepezil loading are shown in Fig. 5. Results confirmed the successful coverage of the AAO membranes with both coatings. For the FTIR data of PMMA coated membranes, the – CH stretching peak appears at 2946 cm^− 1^, while characteristic peaks that appear at 1729, 1142 and 481 cm^− 1^ are related to carbonyl group (C = O) of ester, C-O stretching and C-H bending vibrations, respectively^33,34^. For membranes coated with both PMMA and PU, results showed no significant difference except for the appearance of a very weak peak at 1709 cm^− 1^ which is related to N-H group of PU that appeared as a split from C = O peak, that is seemed to be a result of hydrogen bonding between N-H and C = O groups^35^. Regarding the coated membranes after drug loading, there is no difference except for the intensity of the peaks.

FTIR data related to uncoated AAO membranes before and after drug loading were previously reported^8^, donepezil was shown to exhibit peaks at 2922.75, 2835.6, 1695, and 1587.91 cm^− 1^ while in the present investigation, and these peaks were not prominent since the coating materials could have masked the drug functional groups. As a result, it can be concluded that fabricated AAO membranes are completely covered with the coating materials used to prevent the fouling effect.

#### BET surface area

The obtained results showed that it is worthy to consider that the coating materials can affect the surface area parameters. The highest surface area recorded was related to the AAO membrane (474.55 ± 14.24 m^2^ g^−1^ but the smallest value was for PMMA + PU coating (120.35 ± 3.61 m^2^ g^−1^). The partial infiltration of the polymer into the AAO pores explains the observed decrease in BET surface area after coating. The polymer layer conforms to the pore walls, reducing the effective pore volume and limiting the internal surface that remains accessible to nitrogen adsorption. In addition, narrowing pore entrances further restricts access, collectively resulting in the lower surface area values measured for the coated membranes^36^. Table 1 provides a summary of the surface area characteristics, including BET surface area, pore volume, and pore diameter.

**Table 1:** Surface area parameters determined by BET surface area.

Sample	BET surface area (m^2^ g^−1^)	Pore volume (cm^3^ g^−1^)	Pore diameter (nm)
AAO	474.55 ± 14.24	0.11 ± 0.01	9.08 ± 0.27
PMMA	165.51 ± 4.97	0.71 ± 0.02	17.13 ± 0.51
PMMA + PU	120.35 ± 3.61	0.63 ± 0.02	20.80 ± 0.62

#### Contact angle

**Fig. 6:**
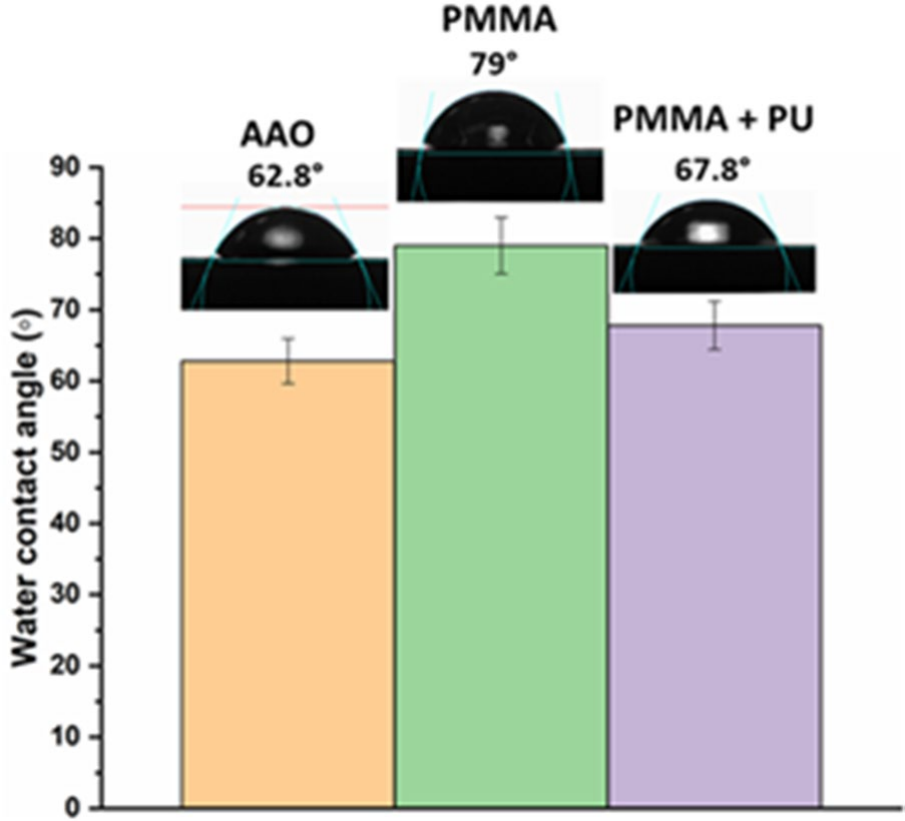
Contact angle values and images of membranes before and after coating.

Contact angle measurement can predict the degree of hydrophilicity and whether surface coatings will minimize fouling effect on the membranes. Results revealed that contact angle of AAO membrane was 62.8° but after coating with PMMA, the contact angle measured 79° indicating increased hydrophobicity. Upon coating with PMMA and PU, the hydrophilicity of the surface was partially lost, compared to uncoated AAO membranes, as the angle slightly increased and measured 67.8° (Fig. 6). It could be explained that the surface hydrophilicity is affected by the surface functional groups, as confirmed by FTIR analysis, which showed an increase in hydrophilic functional groups upon PU addition, which in turn increased the membrane’s hydrophilicity.

### In vitro drug release

**Fig. 7:**
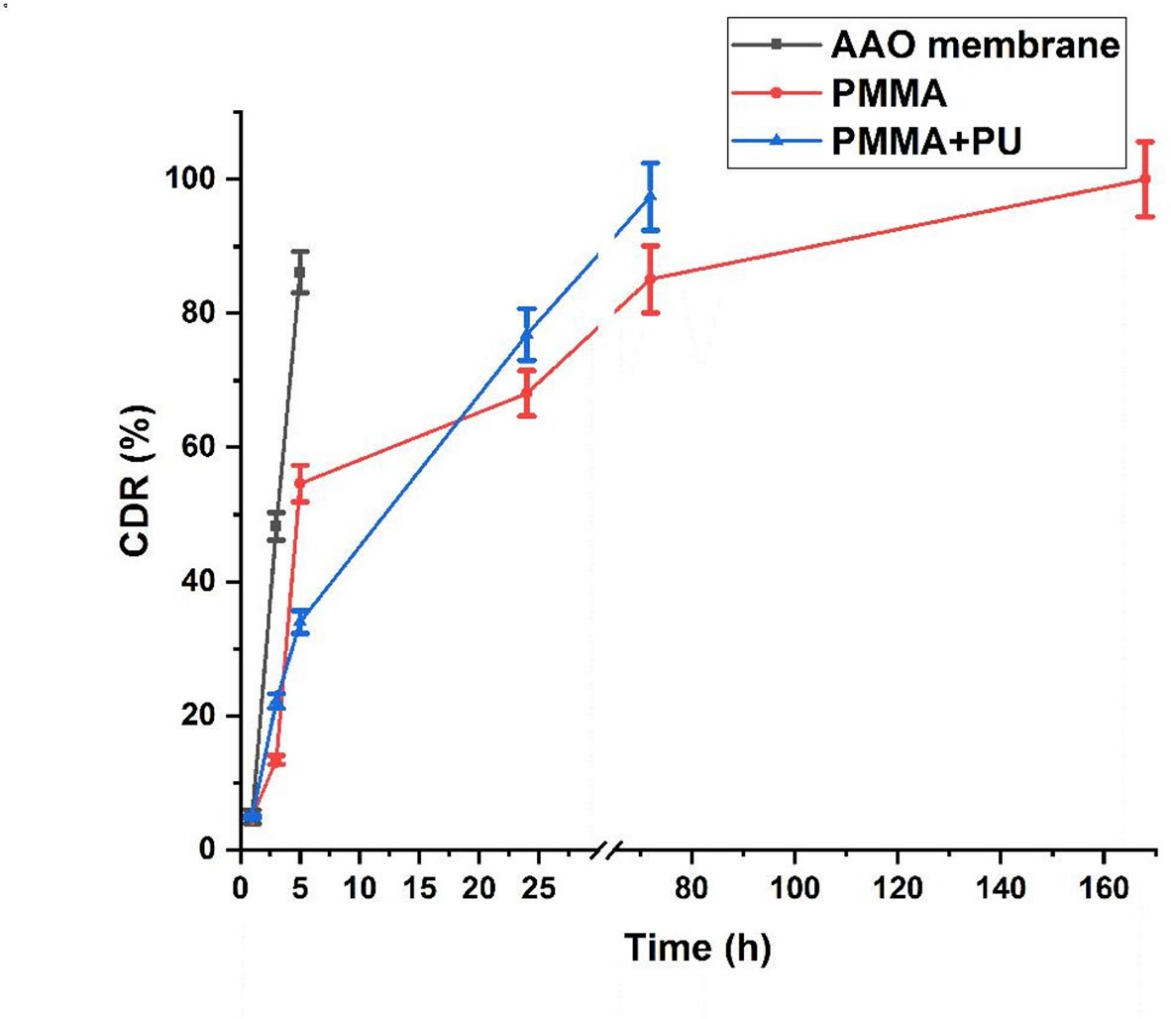
Effect of different coating materials on cumulative donepezil release percentage from AAO membranes.

Cumulative drug release profiles from coated and uncoated AAO membranes are shown in Fig. 7. Within the first 5 h, all membranes showed a burst release with the fastest release rate was observed for uncoated membrane samples, where a rapid initial release (~ 86.14% in 5 h) was observed, suggesting weak drug entrapment and predominance of surface adsorption. This highlights the limitation of the uncoated system for sustained drug release. On the other hand, PMMA + PU samples exhibited a biphasic release pattern characterized by an initial burst release (34% release within the first 5 h) followed by a sustained release phase over 3 days (approximately 97.4% of loaded donepezil was released after 3 days).

Also, PMMA coated AAO membranes showed a gradual increase in drug release rates as the amount of drug released after 3 days was 85%, then the release increased gradually till reached 100% after 7 days, the biphasic release behavior of coated membranes could be explained by the fact that the drug wasn’t completely coated, so in the first hours the release was fast and then slowed down. The derivative release rate (dC/dt) analysis confirmed distinct kinetic behaviors: AAO showed a sharp initial release phase with the highest rate (~ 21.7%/h at 1 h), PMMA exhibited a slower, more gradual release with a modest peak (~ 12.4%/h at 3 h) followed by sustained low rates, while PMMA + PU demonstrated an intermediate biphasic profile with an early peak (~ 8.6%/h at 1 h) and a secondary slower phase extending beyond 24 h (Fig. S5). By analyzing the drug release profiles, it can be observed that coated samples were able to release the drug for a greater period of time than uncoated samples. Consequently, coated AAO membranes may be employed as a regulated localized donepezil delivery system for the management of AD.

### Drug release kinetics

Different mathematical models were applied to donepezil release from AAO membranes and the obtained kinetic parameters are provided in Table 2 and Fig. S6. Mathematical calculations indicated that, the regression coefficient of AAO, PMMA and PMMA + PU was 0.998, 0.64 and 0.80, respectively in zero-order model. On the other hand, according to Higuchi model the regression coefficient was 0.995, 0.796 and 0.93 for AAO, PMMA and PMMA + PU, respectively. Therefore, in the present study, zero-order model was determined to be suitable for defining the kinetics of donepezil release from blank AAO membranes, whereas for PMMA and PMMA + PU, Higuchi model was more suitable.

Based on release kinetic analysis of AAO membranes, zero-order model indicated that the release rate of drug is constant over time which means that the drug is released at a uniform rate until all drug is stripped. Regarding release exponent (*n* = 1.82) where *n* > 1, the release is considered a case II type is governed by erosion or swelling mechanisms^20^. On the other hand, donepezil release from the coated membranes following the Higuchi model of which the rate is directly proportional to the square root of time. The diffusion process across the nanoporous membranes appears to be the main mechanism governing the drug release^37^. Hence, it is suggested that surface coatings affected the behavior of the drug release from nonporous membranes. Diffusion exponent (n) of PMMA and PMMA + PU are 0.54 and 0.66, respectively (*n* < 1), so the release is anomalous (non-fickian).

Based on in vitro obtained data, it can be suggested that PMMA is better used as a coating material than PMMA + PU since it offered a more hydrophobic nature for the drug delivery system and a gradual release of the drug into physiological media. Hence, the in vivo study involved only this membrane.

**Table 2:** Release kinetics parameters of donepezil loaded AAO membranes.

Formula code	Regression coefficient (*R*^2^)^†^			Zero-order model		Higuchi model		Kinetic exponent (*n*)	Maximum release (%)
	Zero-order	Korsmeyer- Peppas	Higuchi	Half-release time (t_50_**) (h)	90% release time (t_90_***) (h)	Half- release time (t_50_**) (h)	90% release time (t_90_***) (h)		
AAO	0.998	0.98	0.995	3.17	5.15	2.93	5.40	1.82	86.14
PMMA	0.64	0.794	0.796	36.11	122.18	21.35	102.04	0.54	100
PMMA + PU	0.80	0.89	0.93	23.52	58.07	15.29	51.75	0.66	97.4

R ^2^ -value is the value of the regression co-efficient, t_50_** is time required for 50% of the drug to be released, t_90_*** is time required for 90% drug release and n is the diffusion exponent.

### In-vivo examination

#### Effect of implants on cognitive functions in AD rat model

**Fig. 8:**
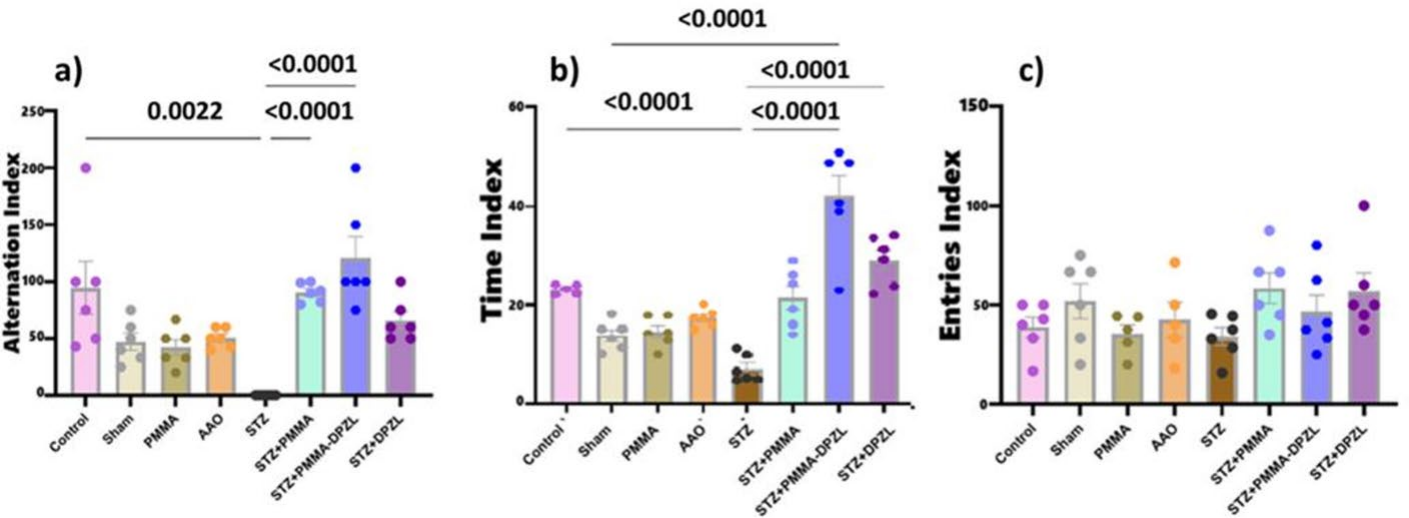
Y-maze performance, (a) spontaneous alternation index, (b) percentage of time spent in novel arm and (c) novel arm entries index. Each group is represented with *n* = 6 rats, data are expressed as mean ± SD, ANOVA followed by Dunn’s multiple comparison test, was employed to determine significance at a confidence level 95% (*p* < 0.05).

AD intracerebral STZ model exhibited many AD pathological features, such as cognitive dysfunction, neuroinflammation, and Aβ deposition. In Y-maze, time index and alternation index showed a significant difference between AD-induced (STZ) and normal control group indicating impaired spatial and working memory after AD induction. The membranes fabricated had no significant effect in normal rats. On the other hand, Implanted membrane (STZ + PMMA), or donepezil-loaded membranes (STZ + PMMA-DPZL) resulted in a significant improvement of time index (21 ± 5.9% and 42 ± 10% vs. 7.1 ± 2.8%) and alternation index (91 ± 8.3% and 121 ± 46% vs. 0, respectively), suggesting cognitive recovery since alternation index can represent the working memory. However, the non-coated membrane loaded with donepezil exhibited no significant difference in comparison to STZ (untreated group) (29 ± 5% time index and 66 ± 19% alternation index), while was not significant from normal groups indicating a partial recovery (Fig. 8a, b). Entries index was not significantly changed in the present study with different treatments (Fig. 8c).

**Fig. 9:**
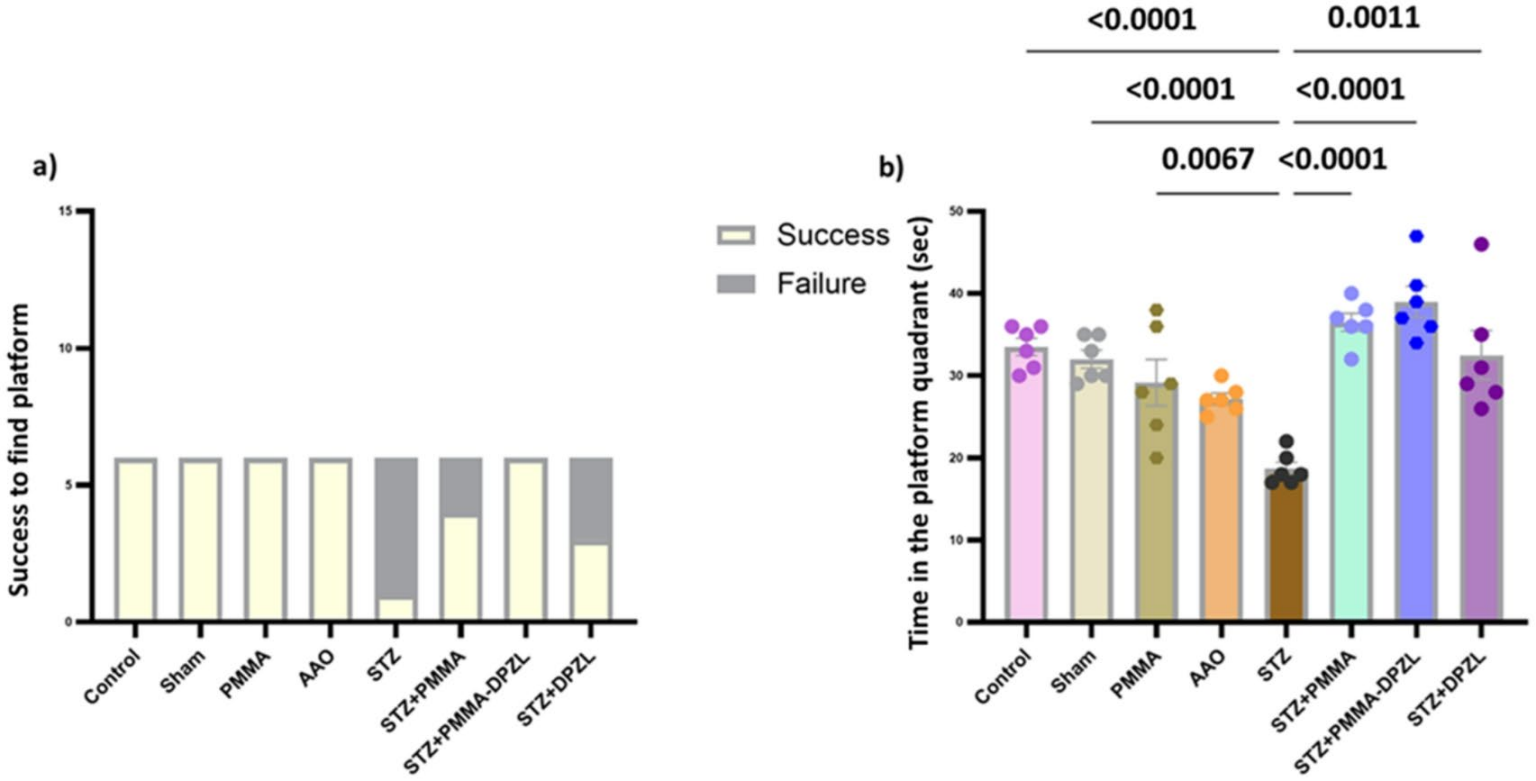
Performance of rats in MWM (a) success and failure trials to reach platform and (b) time spent in platform quadrant. Succes and failure were compared using Chi-Squae model. The MWM target quadrant time is analysed ANOVA followed by Tukey’s multiple comparison test to identify significance at confidence level 95% (*p* < 0.05).

Water maze test is successfully validated for evaluating spatial learning and memory in animal models of neurodegenerative diseases. Results showed that normal rats succeeded to reach the platform, while AD-induced rats (STZ) failed completely (0% success). Results showed that STZ + PMMA and STZ + DPZL groups have enhanced success rates (66.67%, and 50% *p* < 0.05), while STZ + PMMA-DPZL showed no failure rates (100% success) (Fig. 9a). Coated and loaded membranes were of superseded activity. Figure 9b showing the memory retention test, results revealed that control and sham groups spent significantly more time in the platform quadrant confirming good memory performance in comparison with STZ group that showed a significant reduction (control:56.8%, Sham:53.3%, STZ:31.8%, *p* < 0.05). STZ + PMMA, STZ + PMMA-DPZL and STZ + DPZL groups showed an improvement in memory performance (STZ + PMMA:61.8%, STZ + PMMA-DPZL: 65%, STZ + DPZ:55%, *p* < 0.05) (Fig. 9).

**Fig. 10:**
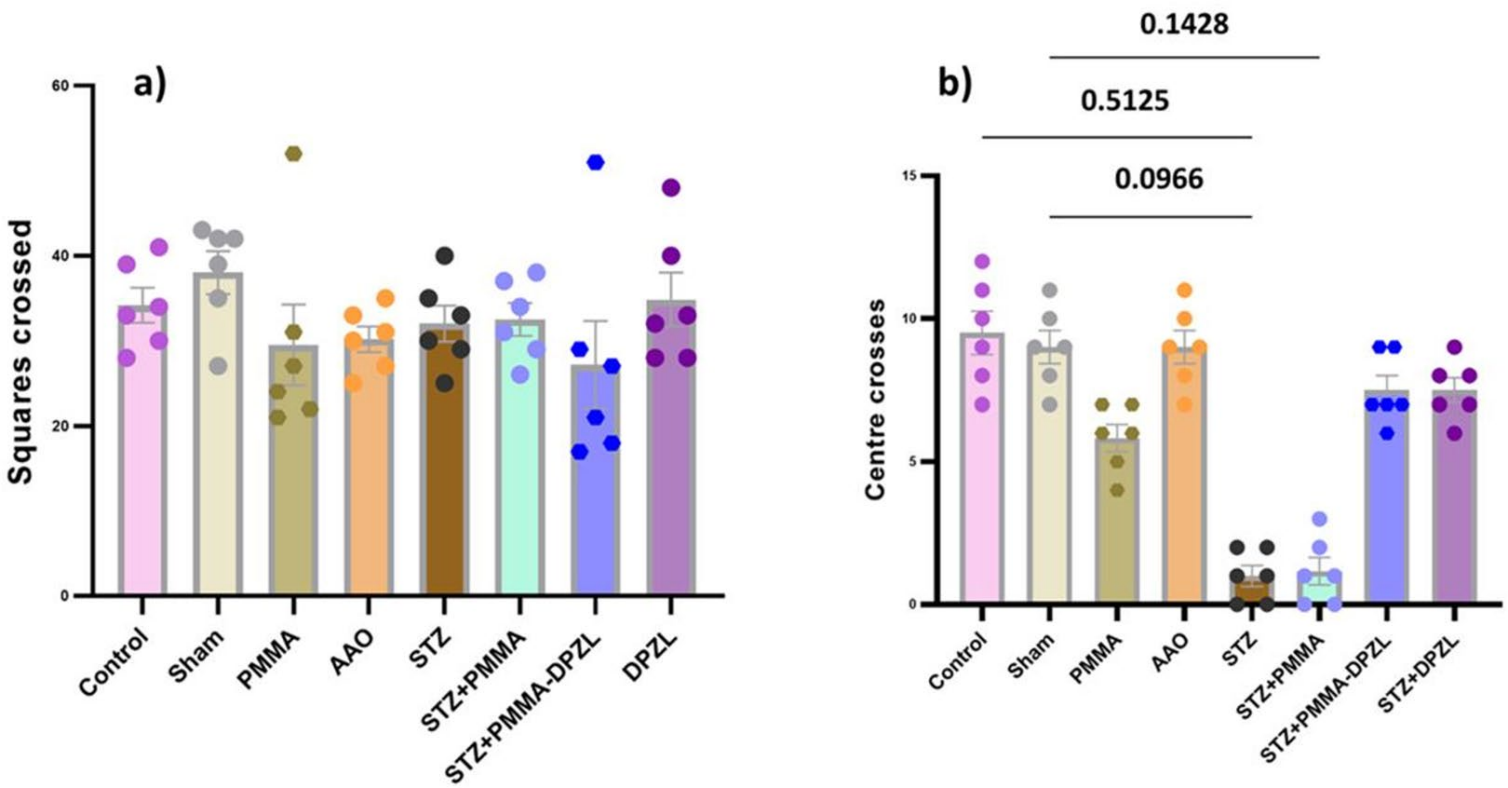
Motor activity of different groups in open field test (a) total number of squares crossed and (b) number of center crosses. Each group is represented with *n* = 6 rats, data are expressed as mean ± SD, ANOVA followed by Dunn’s multiple comparison test was used to identify significance at confidence level 95% (*p* < 0.05).

Number of total crossed squares (Fig. 10a) represents the general locomotor activity of rats, there was no significant difference between different groups indicating absence of signs of motor impairments. the exploratory behavior of rats measured as number of center crosses (Fig. 10b) showed that (STZ) AD-induced rats exhibited a significantly reduced number compared to control or sham groups (1.0 ± 0.89 vs. 9.5 ± 1.9 & 9.0 ± 1.4, *p* < 0.05), which suggests AD-associated anxiety. Similarly, the STZ + PMMA group showed a lower number in comparison to sham group (1.2 ± 1.2) which may be attributed to lack of donepezil action. Other treated AD-induced groups (STZ + PMMA + DPZL, STZ + DPZL) were of ameliorated effect compared to STZ (7.5 ± 1.2 & 7.5 ± 1.0, *p* < 0.05).

**Fig. 11:**
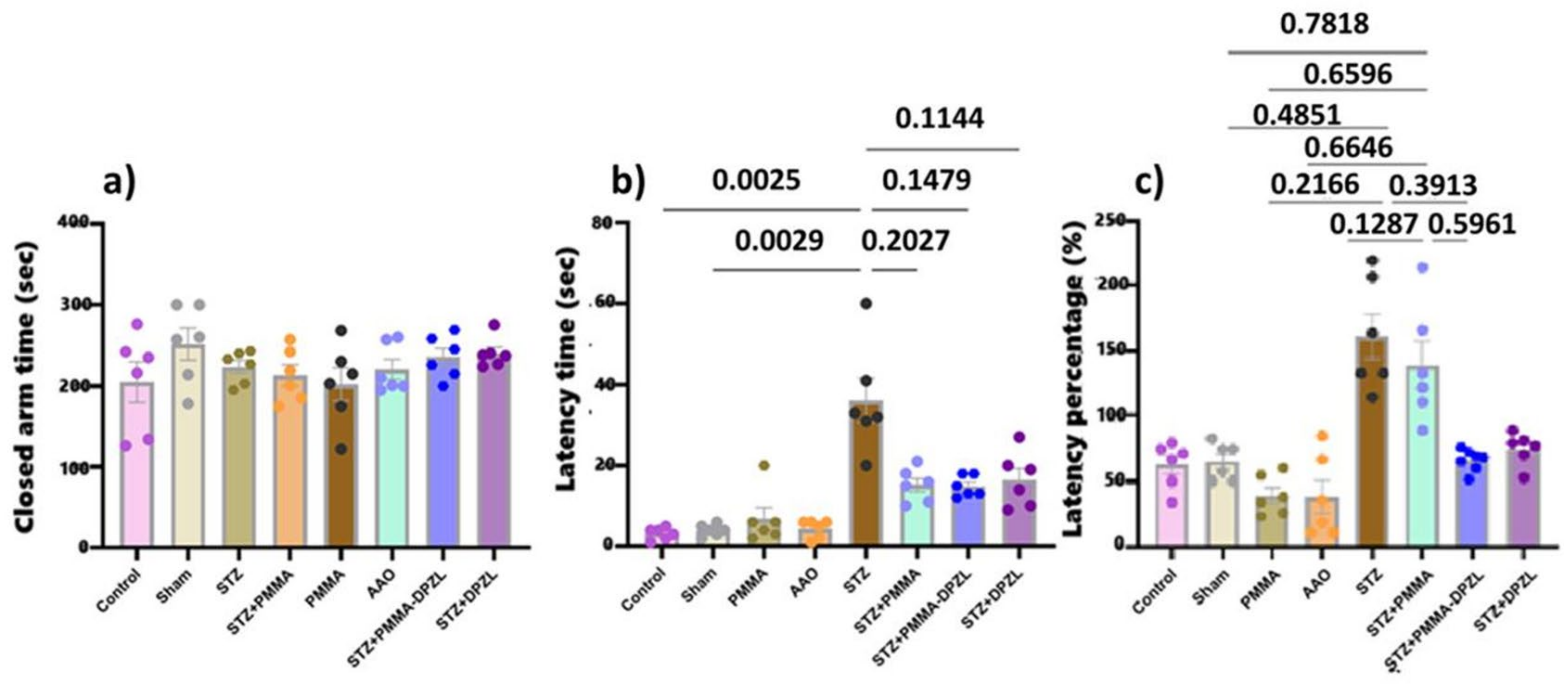
Elevated plus maze performance (a) time spent in closed arm, (b) latency time and (c) latency percentage. Each group is represented with *n* = 6 rats, data are expressed as mean ± SD, ANOVA followed by Tukey’s multiple comparison test was used to identify significance for a and b while Dunn test for c, at confidence level 95% (*p* < 0.05).

Anxiety-related behavior was evaluated using elevated plus maze (Fig. 11). All groups showed no significant difference in time spent in closed arm (Fig. 11a). On the other hand, latency time (Fig. 11b) increased in STZ group (36.0 ± 13.0 s) compared to control, sham (3.2 ± 1.5, 4 ± 1.4 s), PMMA or AAO (6.8 ± 6.6 & 4.3 ± 2.3 s) indicating a shortage of emotional memory. On the other hand, coated membrane treatment of AD-induced rats with or without donepezil loading reduced this latency time (15 ± 4.2, 15 ± 2.6 s, *p* < 0.05). Latency index was shown to be significantly affected by STZ and markedly ameliorated by the membranes implanted (Fig. 11c).

Conclusively, behavioral analyses pointed out that STZ caused an increased anxiety-like behaviour and deficits in learning and memory tasks. Treatment with the PMMA-coated membrane and PMMA-Donepezil loaded membrane induced an enhanced memory performance assessed in Y-maze, MWM and EPM memory retention tests as well as ameliorated associated anxiety presented in open field. The uncoated donepezil-loaded membrane was of a positive effect in the induced model, though was of lower activity than coated ones.

#### Biochemical investigation

##### Acetylcholinesterase activity

**Fig. 12:**
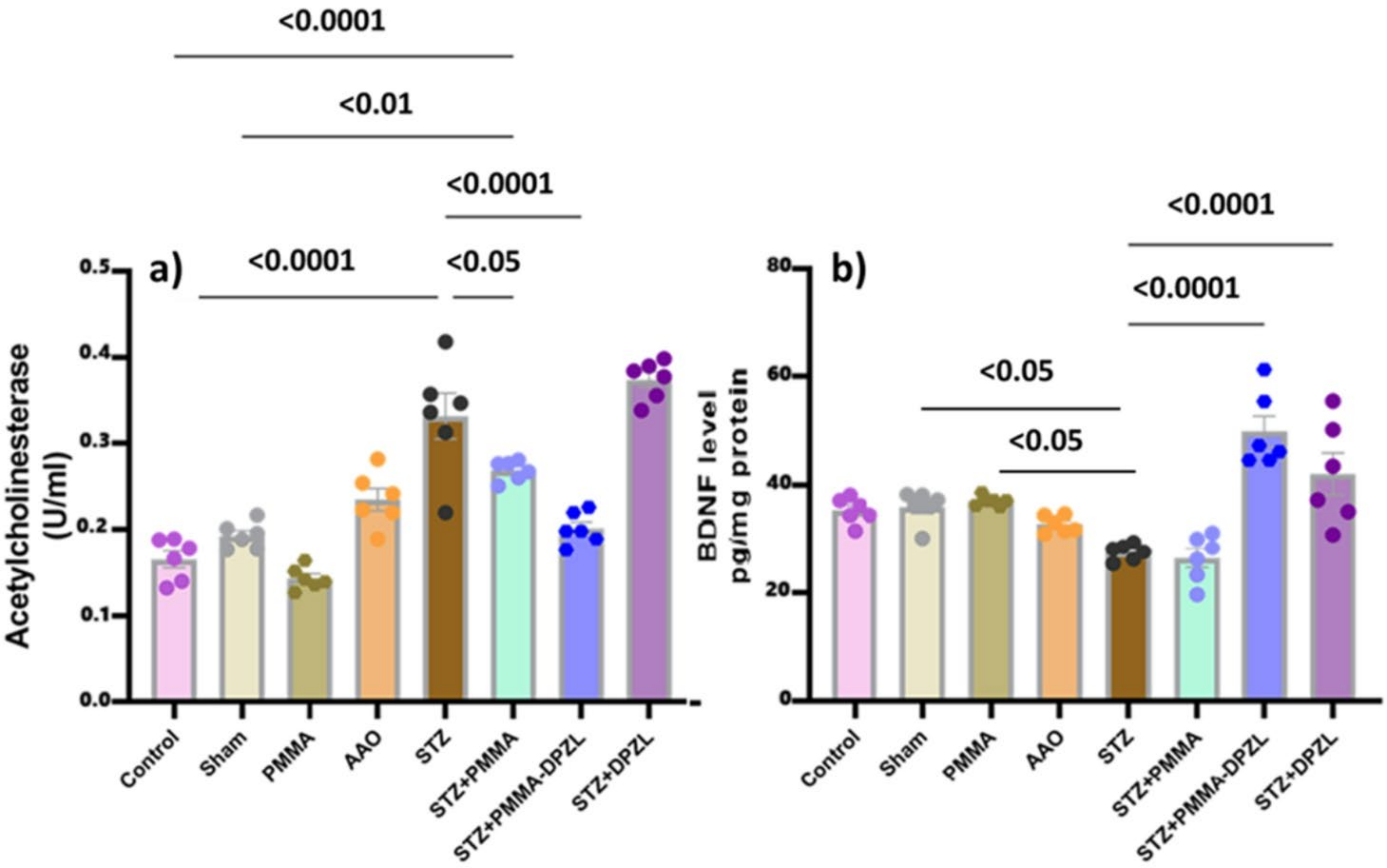
Showing (a) serum acetylcholinesterase (AChE) activity levels and (b) hippocampal BDNF levels. Each group is represented with *n* = 6 rats, data are expressed as mean ± SD, ANOVA followed by Tukey’s multiple comparison test was used to identify significance at confidence level 95% (*p* < 0.05).

STZ has been linked to cholinergic transmission defect which is responsible for the cognitive impairment manifested in the current study as poor performance in MWM and y-maze tests^38,39^. Furthermore, STZ induced AChE enzyme activity compared to control. PMMA-coated membrane implant and the loaded coated membrane (STZ + PMMA-DPZL) were of enhancing cholinergic function but only the loaded and coated membrane could retain normal activity (Fig. 12a). The AchE activity in group implanted with uncoated membrane though loaded with donepezil was not significant from AD-induced group. Donepezil is known as an acetylcholinesterase inhibitor where its activity relies on inhibiting AChE activity to increase the physiological level of Ach which in turn stimulates cognitive functions. The study of Chang Yell Shin et al. (2018) presented a cognitive enhancing effect of donepezil after oral administration to mice at a dose of 3 mg/kg and correlated this effect to a brain level of 45 ng/g which was limited after reaching 100 ng/g. Chang Yell Shin et al. (2018) related the cognitive behavioral improvement to Ach levels in the brain. Other report of donepezil loaded nanoparticles administered at a dose of 15 µg/kg was of cholinergic inducing action in rats and reflected on elevated plus maze and Morris water maze supporting our findings^40,41^.

##### Brain-derived neurotrophic factor (BDNF) assessment

BDNF is a neurotrophic factor essential for neuronal survival, synaptic plasticity, and various cognitive functions. As depicted in Fig. 12b, examination of hippocampal BDNF level in different groups revealed that STZ exhibited the lowest BDNF level that may indicate neurodegeneration. On the other hand, implanting the donepezil-loaded membranes increased the hippocampal BDNF levels (STZ + DPZL: 160.7%, STZ + PMMA-DPZL: 185.7%), while BDNF levels in normal rats were not affected (*p* > 0.05). Indeed, donepezil has been linked to enhanced BDNF levels^42^.

##### Evaluation of hippocampal oxidative stress

**Fig. 13:**
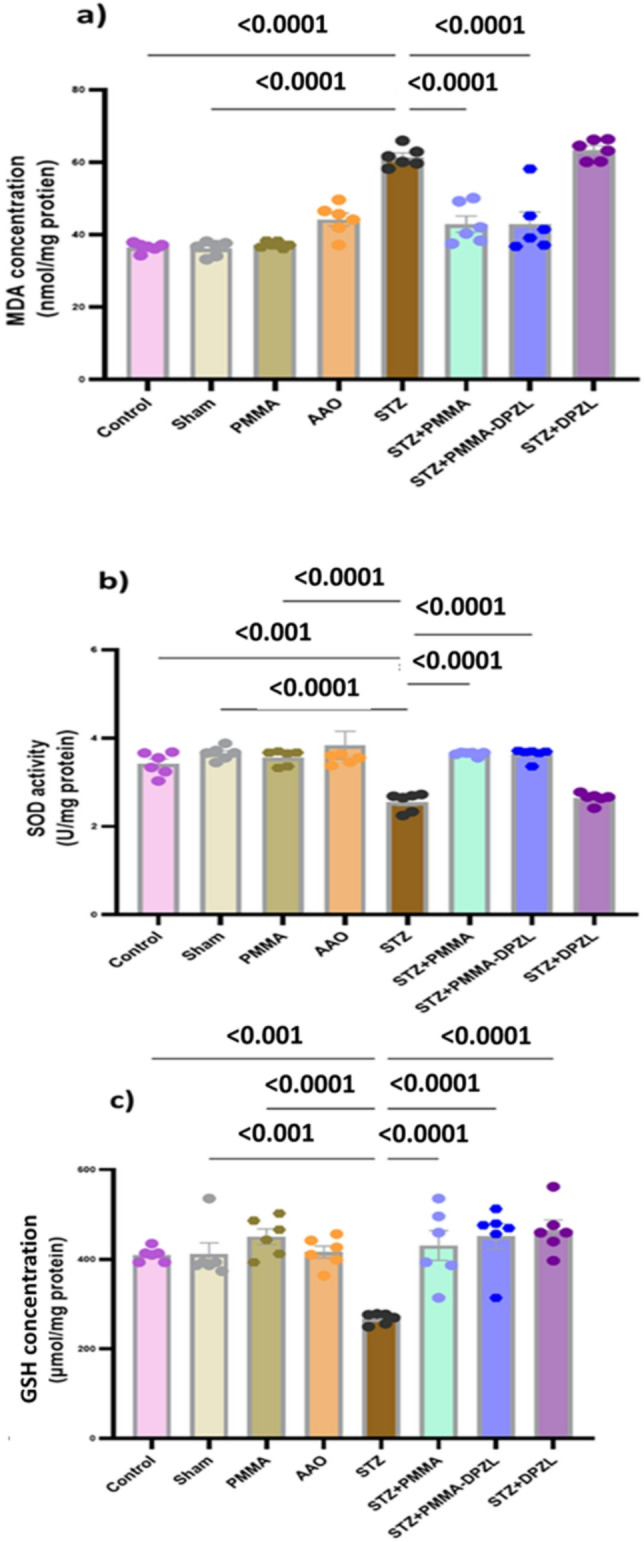
Different oxidative stress and antioxidant markers (a) MDA concentration, (b) SOD activity and (c) GSH content. Each group is represented with *n* = 6 rats, data are expressed as mean ± SD, ANOVA followed by Tukey’s multiple comparison test was used to identify significance at confidence level 95% (*p* < 0.05).

AD-induced by STZ lead to an elevated level of lipid peroxidation marker, MDA compared to control or sham groups (Fig. 13a). Normal rats (control, sham, PMMA and AAO) showed comparable MDA levels, while AD-induced groups were of higher levels (STZ: 61 ± 2.7 nmol/mg protein, *p* < 0.05). Implanting the coated membranes either loaded or non-loaded ameliorated the induced change (STZ + PMMA: 43 ± 5.5 nmol/mg protein, STZ + PMMA-DPZL: 43 ± 8.1 nmol/mg protein, *p* < 0.05), whereas the uncoated membrane showed insignificant change from untreated animals (STZ + DPZL: 63 ± 2.8 nmol/mg protein, *p* > 0.05) (Fig. 13a).

Also, SOD activity was measured and data is represented in Fig. 13b, there is a significant difference between STZ group and all other groups. AD induction using STZ caused a reduction in SOD activity (2.6 ± 0.21 U/mg protein), while, membranes STZ + PMMA, and STZ + PMMA-DPZL, caused a significant increase in SOD activity (STZ + PMMA: 3.6 ± 0.046 U/mg protein, STZ + PMMA-DPZL: 3.6 ± 0.14 U/mg protein) at *p* < 0.05. Regarding the antioxidant GSH, STZ group had lower levels compared to normal ones (STZ: 268 ± 12 µmol/mg protein, Control: 410 ± 15 µmol/mg protein, *p* < 0.05), and STZ + PMMA-DPZL group has the highest level of GSH (451 ± 70 µmol/mg protein, *p* < 0.05) (Fig. 13c).

The biochemical evaluation revealed that STZ increased AChE activity, reduced BDNF levels, while impaired oxidative balance in hippocampus. On the other hand, studied coated membrane either loaded or not with donepezil enhanced these changes and was of comparable results to control animals. The cholinergic hypothesis of AD relates increased cholinesterase activity to deteriorated cognitive function^39^. In accordance, Javed et al., 2015; Liu et al., 2016 and Yamini et al., 2022 showed that ICV-STZ administration increased AChE activity and reported reduced level of ACh at the synapse^38,43,44^. In AD patients, decreased BDNF indicates a lack of trophic support and accelerates neurodegeneration^42^ Generally, AD is linked to deteriorated oxidative state of brain tissue, which is reflected in levels of anti-oxidant enzymes such as GSH, and SOD and pro-oxidant markers such as MDA plays a significant role in the progression of neurodegenerative disorders^38,45,46^.

An interesting observation in the current study is the notion that coated membrane without donepezil drug action could have an ameliorating effect at multiple levels of assays and was of profound behavioral reflection in reducing symptoms of AD. The clearance of β-amyloid from cerebrospinal fluid (CSF) involves a non-enzymatic mechanism wherein interstitial fluid (ISF) flows into the CSF, subsequently following the ISF drainage channel through perivascular basement membranes^47^. Abbott indicated that ISF and CSF can interchange fluid throughout the majority of the ependyma that lines the cerebral ventricles, as well as across the glial layer on the brain’s surface next to the subarachnoid space^48^. It might be suggested that a filtration effect of the membrane coating material could have place and Aβ molecules were trapped out. Removal of β-amyloid from CSF can delay the progression of disease and enhance the brain functions since Aβ deposits activates mitochondrial dysfunction pathways, and release ROS, to induce an apoptotic cascade^49–51^. PMMA dialyzers showed adsorption-based clearance of similar structures such as macroglobulin or inflammatory cytokines in addition to diffusion coupled mechanism. The membrane showed a significant reduction of amyloidosis and inflammation in renally impaired patients^52–54^.

Furthermore, donepezil delivery to brain tissue was of ameliorative function in support with previous literature^55,56^. This drug delivery model can be of an added value for kidney or liver function impaired patients as very minute doses are required and applied directly in the CSF offering a safe alternative. Use of unloaded membranes prevent any side effects related to drugs on long term. However, a limitation of the study include small sample size investigated and the inability to detect β-amyloid in the implants after removal from animals at the end of study. Future studies should focus on the application of Safe CSF devices for such chronic neurodegenerative diseases.

#### Histological study of hippocampus

**Fig. 14:**
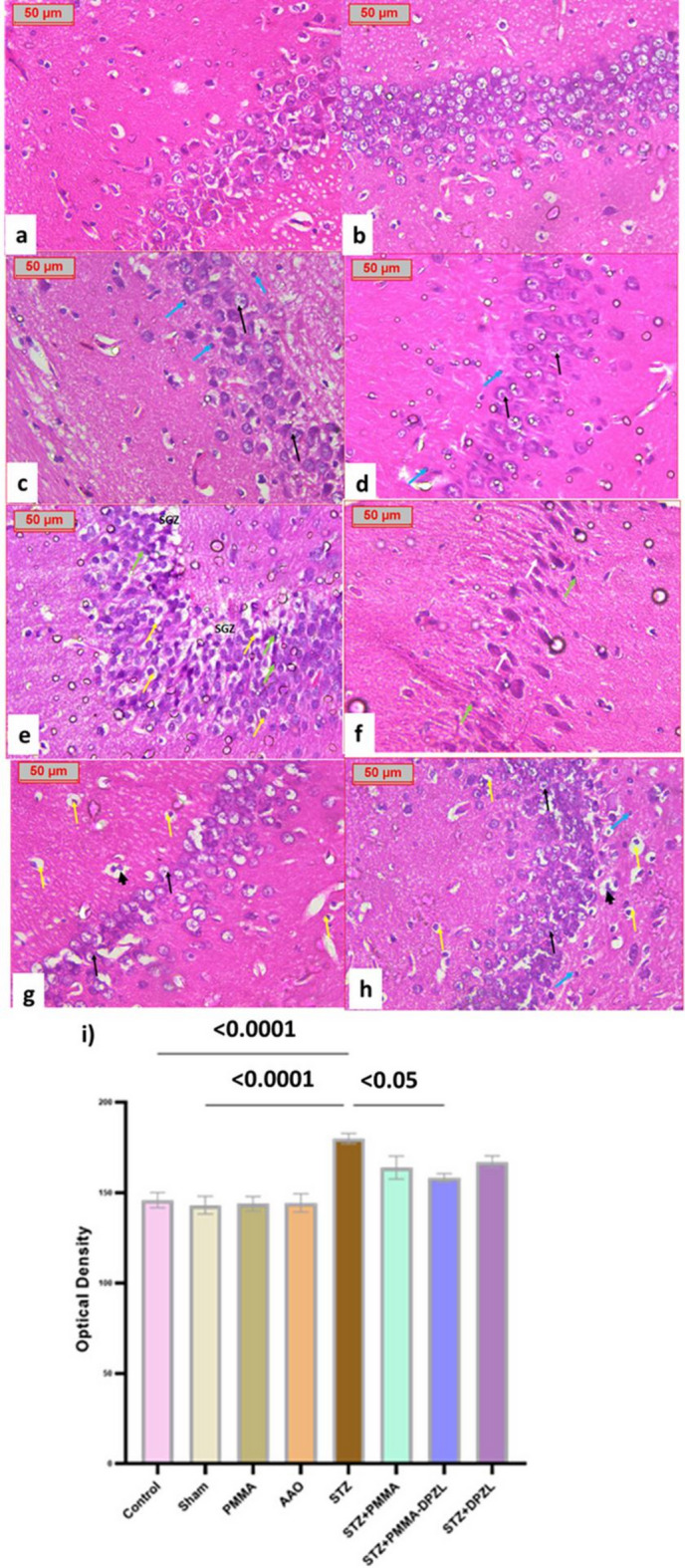
Histopathological photomicrographs of hippocampus tissue sections stained with hematoxylin and eosin (H&E x400). (a, b) control and sham groups showing normal hippocampal architecture with intact neuronal structure; (c) PMMA implanted rats displaying well-organized pyramidal neurons with normal histology, large vesicular neuronal nuclei (black arrow), and glial cell nuclei (blue arrow); d) AAO group showing hippocampus of moderately organized pyramidal cells with normal histology, normal neuronal cells with large vesicular (black arrow) stained nuclei of glial cells (blue arrow) and mild congestion e) STZ group showing disrupted and loosely packed pyramidal neurons, widespread neurodegeneration with pyknotic nuclei (green arrow), pericellular spaces (yellow arrow), and widened SGZ area; f) STZ + PMMA group exhibiting similar degenerative changes including flame-shaped neurons (white arrow) and pyknotic nuclei (green arrow); g) STZ + PMMA-DPZL group showing well-preserved hippocampal structure, with closely packed neurons (black arrow), limited neurodegeneration (yellow arrow), and few immature neurons (short arrow); h) STZ + DPZL group demonstrating moderate restoration of pyramidal cells with evidence of both normal neurons (black arrow), neurodegeneration (yellow arrow), glial nuclei (blue arrow), and immature neurons (short arrow) and i) Optical density of congo red stained brain tissue. Data are extracted from 5 different tissue sections and expressed as mean ± SD, ANOVA followed by Tukey’s multiple comparison test was used to identify significance at confidence level 95% (*p* < 0.05).

The histological analysis of hippocampus revealed no signs of neurodegeneration in control, sham, PMMA and AAO groups despite some congestion and distortion of neuronal organization in PMMA- and AAO-implanted rats that may be attributed to membrane compression (Fig. 14a-d). STZ and STZ + PMMA groups showed severe neurodegenerative changes and Alzheimer’s disease pathology hallmarks (Fig. 14e, f). The presence of donepezil with PMMA coating showed a neuronal integrity and some immature neurons were observed (Fig. 14g), On the other hand, STZ + DPZL group showed partial restoration of neuronal structure (Fig. 14h). Congo red positive β-amyloid stained deposits were significantly exhibited after STZ injection *p* < 0.05, while it was markedly reduced in the loaded-coated membrane treated animals, *p* < 0.05. On the contrary, other treatments were of comparable staining to STZ group (Fig. 14i).

The current investigation showed degenerative changes in STZ-induced group with pyknotic nuclei and scattered amyloid plaques, neurofibrillary tangles were observed while the applied membranes showed mild congestion that could be linked to the exerted pressure from the membrane placement. Nevertheless, the hippocampal tissue showed normal structures in loaded membrane-implanted animals.

## Conclusion

The study successfully created the AAO nanoporous membrane coated with PMMA, which serves as a novel intracerebral drug delivery system for donepezil in AD treatment. The PMMA coating ensured continuous docile release in seven days, while the uncoated membranes demonstrated fast release. In an STZ-induced AD rat model, these membranes demonstrated efficacy in cognitive tests, reduced neuroinflammation and preserved neuronal acetylcholinesterase activity, improved BDNF level and antioxidant balance. This localized delivery system provides a transformative approach to the treatment of AD, especially for patients unable to bear oral drugs, and suggests dual medical benefits through potential β-amyloid filtration. Overall, these findings pave the way for individual, transplantable drug delivery devices that increase the quality of life for AD patients with targeted, long-term medical effects.

## Limitations

Limitations of the presented study include small sample size investigated, the lack of β-amyloid determination in the explants at the end of study, short time of release and the use of a specific model. Future studies should assess long-term biocompatibility, larger cohorts, and alternative AD models (e.g., transgenic mice) to address the application of CSF devices for such chronic neurodegenerative diseases.

## Supplementary Information

Below is the link to the electronic supplementary material.

Supplementary Material 1

## Data Availability

The datasets used and/or analysed during the current study available from the corresponding author on reasonable request.
