# Supplementary material for: Modulation of experimental Alzheimer’s disease in rats through donepezil-loaded CSF implant
Source: Sci Rep. 2025 Nov 25;15:42017. doi: 10.1038/s41598-025-26181-z (PMC12657875; doi:10.1038/s41598-025-26181-z)
Supplement: Supplementary file 1 — Supplementary Material 1 [file 41598_2025_26181_MOESM1_ESM.pdf]

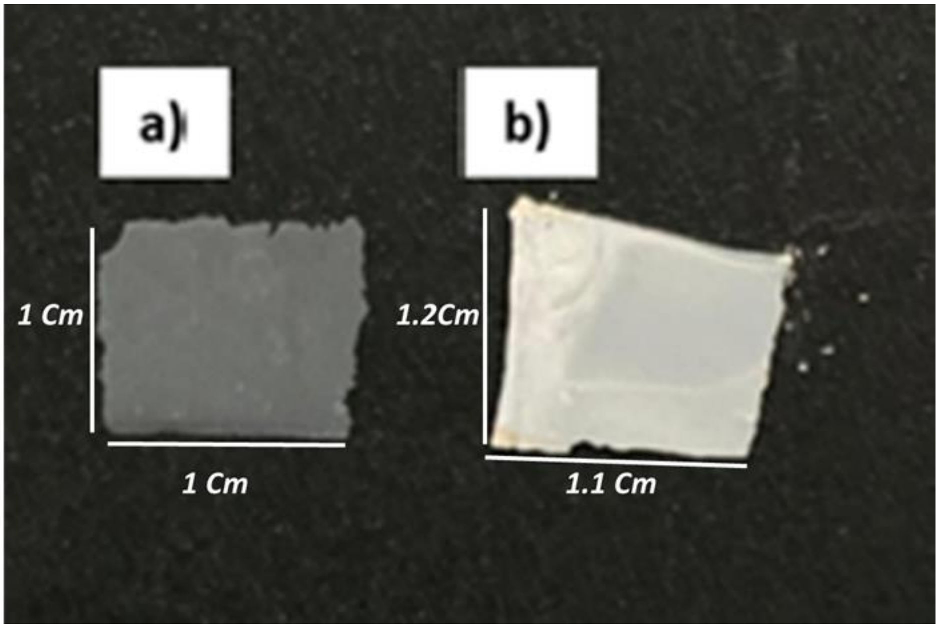

Figure S1: Visual images of a) uncoated AAO membrane and b) coated membrane.

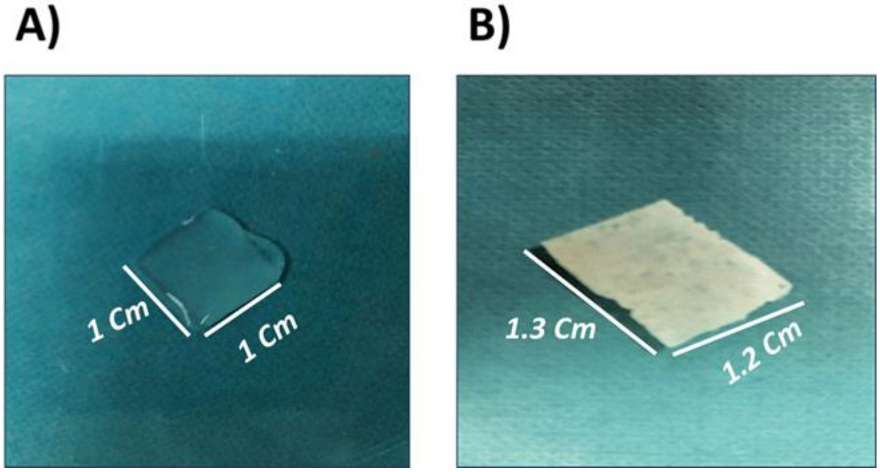

Figure S2: Visual images of a) drug loading process and b) membrane after drug loading and surface coating.

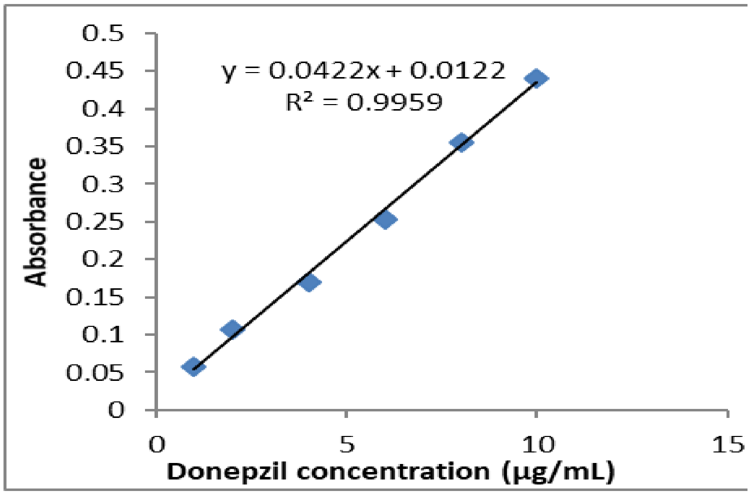

Figure S3: Calibration curve of donepezil HCl with the corresponding linear regression equation and correlation coefficient (R^2).

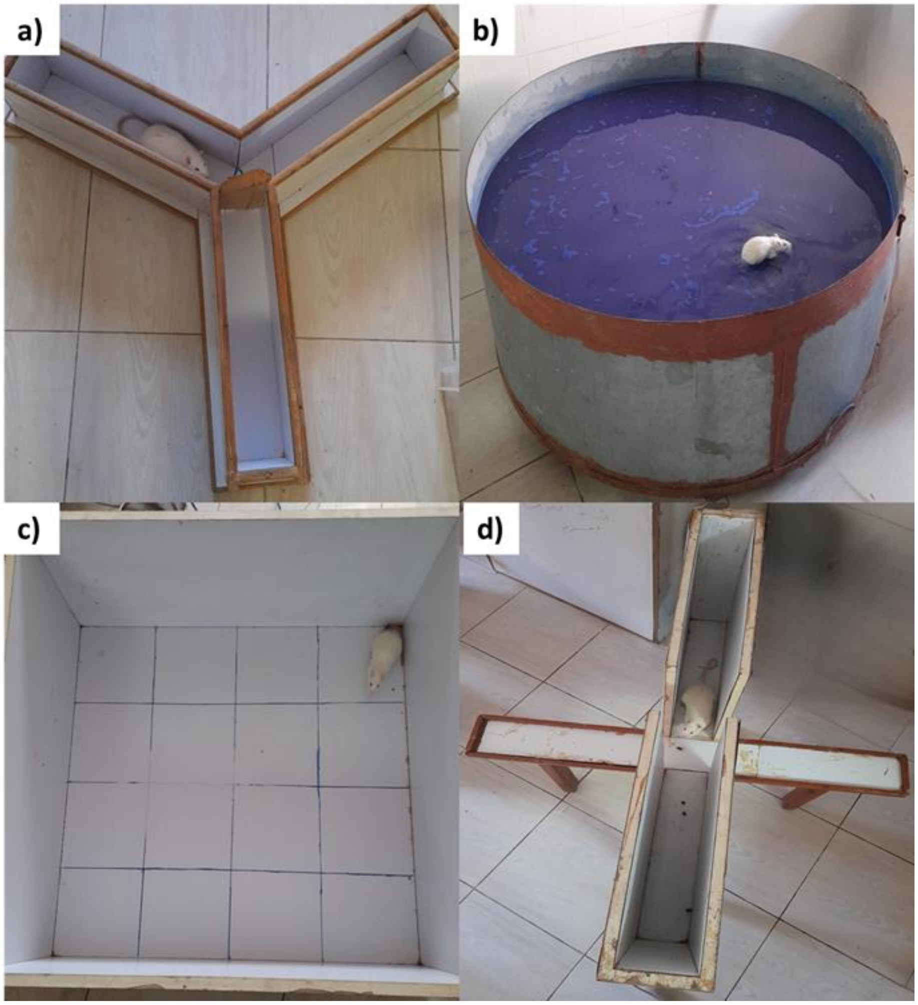

Figure S4: behavioral analyses for rats a) Y-maze, b) Morris water maze, c) open field and d) elevated-plus maze.

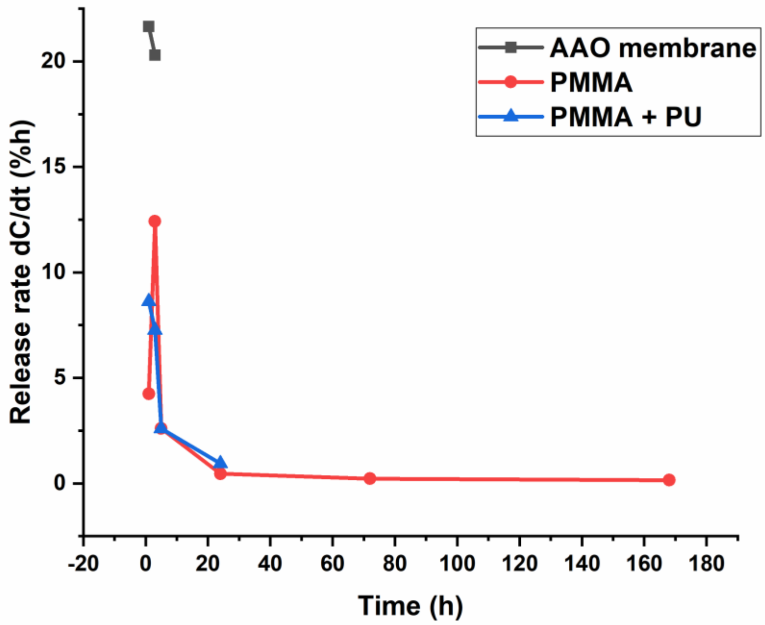

Figure S5: Spontaneous drug release rate (dC/dt) profiles of uncoated and coated samples.

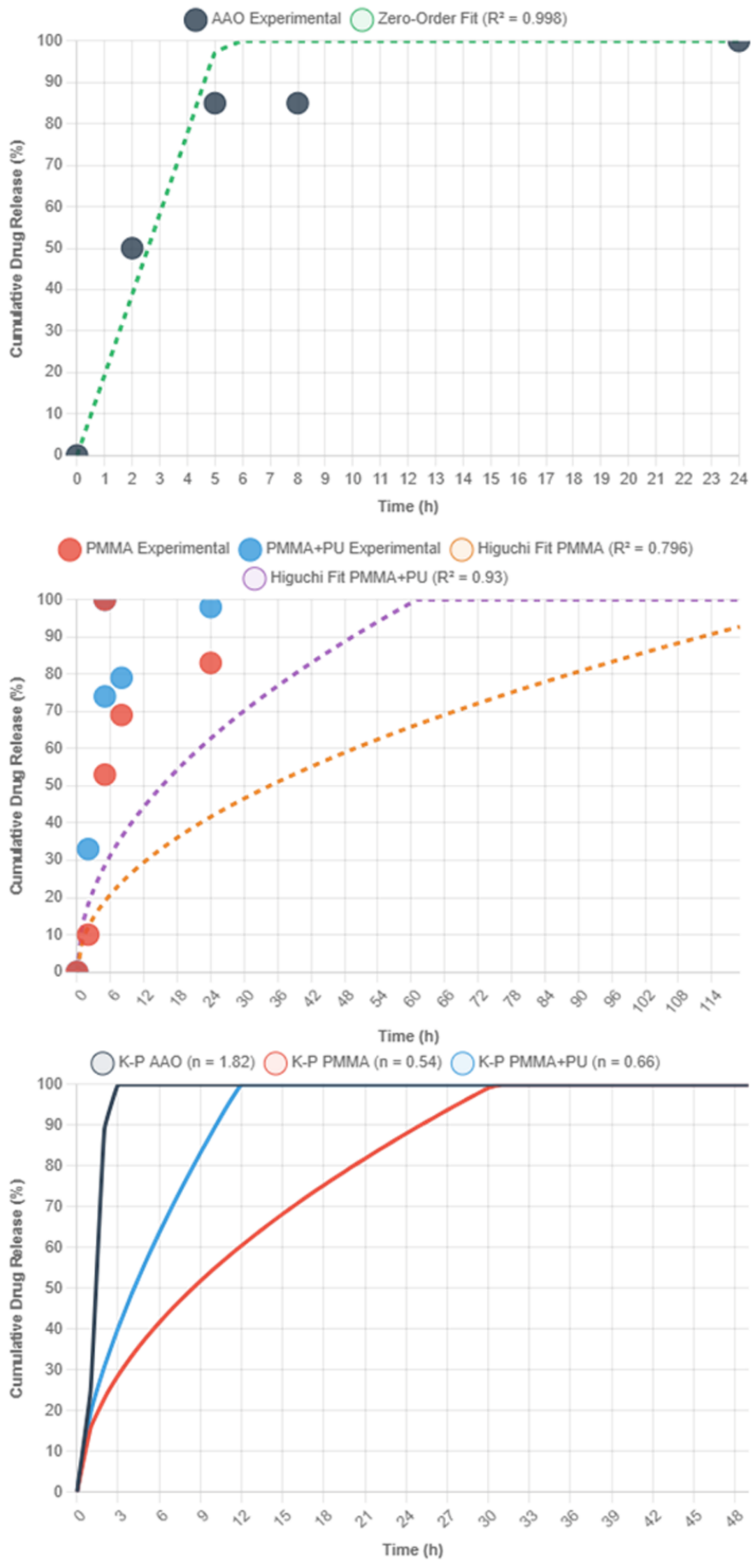

Figure S6: Drug release models a) zero-order, b) Higuchi and c) Krosmeyer-peppas
